# Clustering of health-related behaviours within children aged 11–16: a systematic review

**DOI:** 10.1186/s12889-020-10140-6

**Published:** 2021-01-14

**Authors:** Victoria Whitaker, Melissa Oldham, Jennifer Boyd, Hannah Fairbrother, Penny Curtis, Petra Meier, John Holmes

**Affiliations:** 1 Health Sciences School, University of Sheffield, Sheffield, UK; 2 School of Health and Related Research, University of Sheffield, Sheffield, UK

**Keywords:** Cluster analysis, Health behaviours, Youth, Multiple risk factors, Systematic review, Children

## Abstract

**Objective:**

We aimed to systematically review and synthesise evidence on the clustering of a broad range of health-related behaviours amongst 11–16 year olds.

**Method:**

A literature search was conducted in September 2019. Studies were included if they used cluster analysis, latent class analysis, prevalence odds ratios, principal component analysis or factor analysis, and considered at least three health-related behaviours of interest among 11–16 year olds in high-income countries. Health-related behaviours of interest were substance use (alcohol, cigarettes and other drug use) and other behavioural risk indicators (diet, physical activity, gambling and sexual activity).

**Results:**

The review identified 41 studies, which reported 198 clusters of health-related behaviours of interest. The behaviours of interest reported within clusters were used to define eight behavioural archetypes. Some included studies only explored substance use, while others considered substance use and/or other health-related behaviours. Consequently, three archetypes were comprised by clusters reporting substance use behaviours alone. The archetypes were: (1) Poly-Substance Users, (2) Single Substance Users, (3) Substance Abstainers, (4) Substance Users with No/Low Behavioural Risk Indicators, (5) Substance Abstainers with Behavioural Risk Indicators, (6) Complex Configurations, (7) Overall Unhealthy and (8) Overall Healthy.

**Conclusion:**

Studies of youth health behavioural clustering typically find both a ‘healthy’ cluster and an ‘unhealthy’ cluster. Unhealthy clusters are often characterised by poly-substance use. Our approach to synthesising cluster analyses may offer a means of navigating the heterogeneity of method, measures and behaviours of interest in this literature.

**Supplementary Information:**

The online version contains supplementary material available at 10.1186/s12889-020-10140-6.

## Introduction

The clustering of health behaviours has important consequences for health as the risks associated with engagement in any particular behaviour may increase, or decrease, depending on which other behaviours an individual engages in [1]. Where behaviours do cluster, multi-behavioural prevention and health promotion strategies may also be more effective than those targeting a single behaviour. Similarly, the effectiveness of efforts targeting one behaviour in isolation may vary depending on which other behaviours individuals’ engage in [1].

Analyses of the clustering of health behaviours are interested in whether individuals participate in each of a set of health behaviours and whether an exhaustive set of ‘clusters’ or ‘behavioural types’ can summarise the patterns of participation seen across a population [2]. For example, three clusters may broadly summarise the patterns of participation in a population: individuals either (i) smoke, drink heavily, and use illicit drugs; (ii) drink heavily; (iii) do none of these behaviours. Analyses of clustering investigate underlying associations between concurrent behaviour [2] and they seek to exhaustively classify patterns of behaviour across the whole population rather than describing patterns in one part of the population (e.g. the tendency for illicit drug users to also smoke).

Clustered patterns of health-related behaviour often emerge in adolescence [3–6], and clusters involving multiple adverse health-related behaviours have been found to be more prevalent amongst younger adults than in older age groups [7]. A 2006 review of health-related behaviours among young people considered the relationships between alcohol, smoking, safe sex, and dietary behaviours amongst 10–18 year olds [8]. The authors found extensive evidence that smoking and alcohol consumption cluster within individuals and, to a lesser extent, found clustering of alcohol consumption, smoking and risky sexual behaviour. More recent reviews [7, 9] have focused on adult populations. In these reviews, both ‘healthy’ and ‘non-risky’ clusters were common: such clusters were characterised by low, or no, participant engagement in the risk behaviours considered by studies [7, 9]. Polarisation was also apparent: primary studies often reported engagement by some participants in all, or none, of the health-related behaviours measured [7, 9].

In addition to a lack of recent reviews of the clustering literature for adolescents, there are a number of other limitations within current evidence. First, the extent to which reviews are able to compare behavioural clusters is limited by significant heterogeneity between primary studies. Such heterogeneity is apparent in terms of the measures used, and the statistical analysis techniques employed (the sensitivity of those techniques to small variations in the data [2]). Reviews to date have not addressed this directly, tending to focus elucidating the behaviours that consistently cluster between studies [7, 9]. Second, although many studies examine clustering of diet, physical activity, alcohol consumption and smoking; other behaviours, such as risky sexual behaviour and gambling, are given less attention [10–12]. Moreover, health-related behaviours that are emerging as areas of concern for health, such as overuse of internet-based technologies [12, 13], are not addressed at all. Third, explorations of how health-enhancing behaviours relate to health-compromising behaviours, is limited [8].

Given these limitations, this study aims to systematically review the literature on the clustering of a broad range of health-related behaviours amongst 11–16 year olds. A secondary aim, is to identify a method for synthesising highly heterogeneous results from clustering studies.

## Methodology

### Literature search

We searched the MEDLINE, CINAHL and PsychINFO databases on 24th September 2019.

Terms relating to four areas (analytical method, adolescents, health-related behaviour(s) as a general concept, and specific health-related behaviours such as alcohol use) informed a combination of free text and MESH search terms (see Supplementary Table 3 for full search strategy). Methodological terms were selected to identify analyses of the clustering of multiple behaviours [2, 9] rather than analyses of the co-occurrence of two behaviours (e.g. bivariate correlations). No time limits were imposed on the search. The study protocol was not preregistered.

### Inclusion and exclusion criteria

Included studies were from high income countries (identified in relation to World Bank criteria) to increase comparability of findings. Informed by recent youth health behavioural trends that have been limited to high income countries [14], we reasoned that differences in the lives and health behaviours of young people between high and low income countries may be substantive. We defined studies of clustering as primary studies using any of the following analytical methods: cluster analysis, latent class analysis, prevalence odds ratios, principal component analysis, and factor analysis.

We initially planned to review studies of 11–24 year-olds, but narrowed this to 11–16 year-olds after completing study selection due to the number of eligible studies identified and the heterogeneity of the age groups studies and the clusters identified within those studies. Data were typically from school surveys of 15 year olds and younger (e.g. the Health Behaviours in School Children survey or the European School Survey Project on Alcohol and other Drugs), or of adults aged 18 years and above. Therefore, to reduce methodological heterogeneity across our included studies, we screened titles and abstracts for samples aged 11–24 and then screened full papers for samples aged 11 up to and including 16 years. Studies reporting data from a sample with a wider age range than 11–16 years were included if it could be determined that 50% or more of the sample were aged 11–16 years or that the mean age was 16.

We initially defined eight key health-related behavioural areas of interest: alcohol consumption, tobacco smoking, cannabis use, other illicit drug use, sexual activity, physical activity, dietary behaviours, and internet-based technology use. However, although there is increasing concern about the health and social risks associated with adolescents’ use of internet-based technologies, evidence increasingly suggests it is the mode, pattern or extent of use, not use per se, that is problematic [12]. Our initial searches revealed these aspects of use are not well-measured in the available literature and we subsequently removed internet-based technology use from our behaviours of interest to avoid weakening the analysis. Behavioural areas of interest ranged in their scope: some encompassed a single behaviour (e.g. smoking), while others, such as drug use, encompassed multiple behaviours (e.g. cocaine use, cannabis use). Consequently, included studies were required to analyse the clustering of at least three health-related behaviours across two or more of the behavioural areas of interest (e.g. studies examining alcohol drinking, heroin use and cocaine use were permissible as this covers two areas; those examining heroin, cocaine and cannabis use were not as this is a single area – drug use).

Analyses employing cluster transition analyses were excluded as we wished to establish the composition of behavioural clusters at a given time, rather than the pathways between behavioural clusters over time. Studies with vulnerable populations were also excluded to increase the comparability of findings. A vulnerable group was defined in relation to whether the group in question would be expected to be associated with particular groups of risky health behaviours or social marginalisation. For example, young people in the youth justice system exhibit elevated levels of substance use [15]. We acknowledge the limitations of this approach in the discussion.

### Paper screening and data extraction

Two authors (VW and MO) screened paper titles and abstracts. Four, separate, random subsamples of 100 titles and abstracts (400 in total) were double coded and Cronbach’s alpha was used after each subsample to measure internal consistency. Chronologically, the results were: 0.46 (fair agreement), 0.69 (good agreement), 0.53 (fair agreement) and 1.00 (excellent agreement). The lower agreement in early subsamples reflects a lack of clarity in many titles and abstracts regarding the analytical methods used. Disagreement was overcome through group discussion and analysis of the full text.

Data extraction was undertaken by MO, VW, JB, JH, and HF. For the purposes of the analysis presented in this paper, data pertaining to the age, ethnicity, and gender of participants, the behavioural clusters identified by each study, and the geographical origin of the study were extracted. Quality appraisal of individual studies was conducted by JB using the AXIS critical appraisal tool [16]. MO double appraised studies to check for agreement.

Analyses of behavioural clusters generate a large number of numerical results and different analytical methods produce different metrics. To aid comparison of data during synthesis, we converted the primary study results into prose using a protocol agreed between the data extractors. Specifically, we converted probabilities and factor loadings into the following language: No = < 5% (or < 0.05), Very unlikely = 5 - < 15%, Unlikely = 15 - < 35%, May = 35 - < 65%, Likely = 65 - < 85%, Very likely = 85 - < 95%, All = 95%+. Where analyses provided mean scores rather than probabilities (e.g. in cluster analyses), data extractors compared the scores across clusters to decide whether they were reflective of low, medium or high on measures of different behaviours. For example, in an instance where there were 3 clusters which scored a mean of 1, 5 and 10 respectively on a measure, the first would be considered low, the second medium and the third high.

### Synthesis of clusters from included studies

Existing guidance for synthesising findings from reviews of clustering analyses is limited, we therefore followed Noble et al. [9] by tabulating which of our seven behaviours of interest were measured by each primary study. Next, we calculated the percentage of studies by the numbers and combinations of our behaviours of interest that they measured. However, we also required a method to group together clusters with apparently similar behavioural patterns identified in different studies. Through group discussion, we developed a new iterative approach that involved organising clusters into ‘archetypes’.

The process for constructing the archetypes is summarised below. Unlike previous reviews [7, 9], this relied solely on the behaviours measured and the patterns of engagement in behaviours reported within clusters. Cluster titles provide a poor basis for comparison between studies as they are often informed by the topic foci of individual studies, which were highly varied. While titles akin to ‘substance users’ were common, the measures used to define substance use were similarly varied between studies. Also, titles often referred to behaviours that were included in the analyses of an individual study, but which were outside of the scope of our review (e.g. substance using bullies). Cluster titles did not therefore inform the construction of archetypes. Our process was as follows:

1. Extract a description of all clusters identified in the included primary studies using consistent natural language to describe patterns of engagement in behaviours reported within clusters.
2. Develop an initial set of archetypes by grouping together clusters involving similar behaviours and patterns of engagement in those behaviours.
3. Refine this initial set iteratively through discussion and consensus within the research team.
4. Produce a written description of each archetype, including a name and inclusion criteria and check all constituent clusters fit this description.
5. Discuss and resolve difficult cases that do not clearly fit within archetypes, refining archetype descriptions as necessary.
6. Review archetypes for parsimony by, for example, renaming, aggregating or disaggregating them.
7. Analyse the clusters to inform a narrative synthesis, giving particular attention to the number and key characteristics of each archetype’s constituent clusters.

Our archetypes were defined only in relation to the seven behavioural areas of interest discussed above and not with reference to other behaviours included (e.g. bullying, sleep). The seven behavioural areas were split into two categories to enable meaningful synthesis, namely: substance use (alcohol, tobacco and other drug use) and other behavioural risk indicators (diet, physical activity, gambling and sex). While some studies included measures of protected and unprotected sex, all but two samples [17, 18] included children younger than the age of consent in the country of interest in the study sample. As most papers ran clustering analyses on the full sample, disaggregation of results by age were not possible. We therefore took a conservative approach and categorised any sexual activity as a negative risk indicator. Following Delk et al. [19], we treated e-cigarette use and tobacco smoking as use of the same substance. Cannabis and synthetic cannabis were also treated as a single substance, as in Lee et al. [20].

## Results

### Search results

**Fig. 1:**
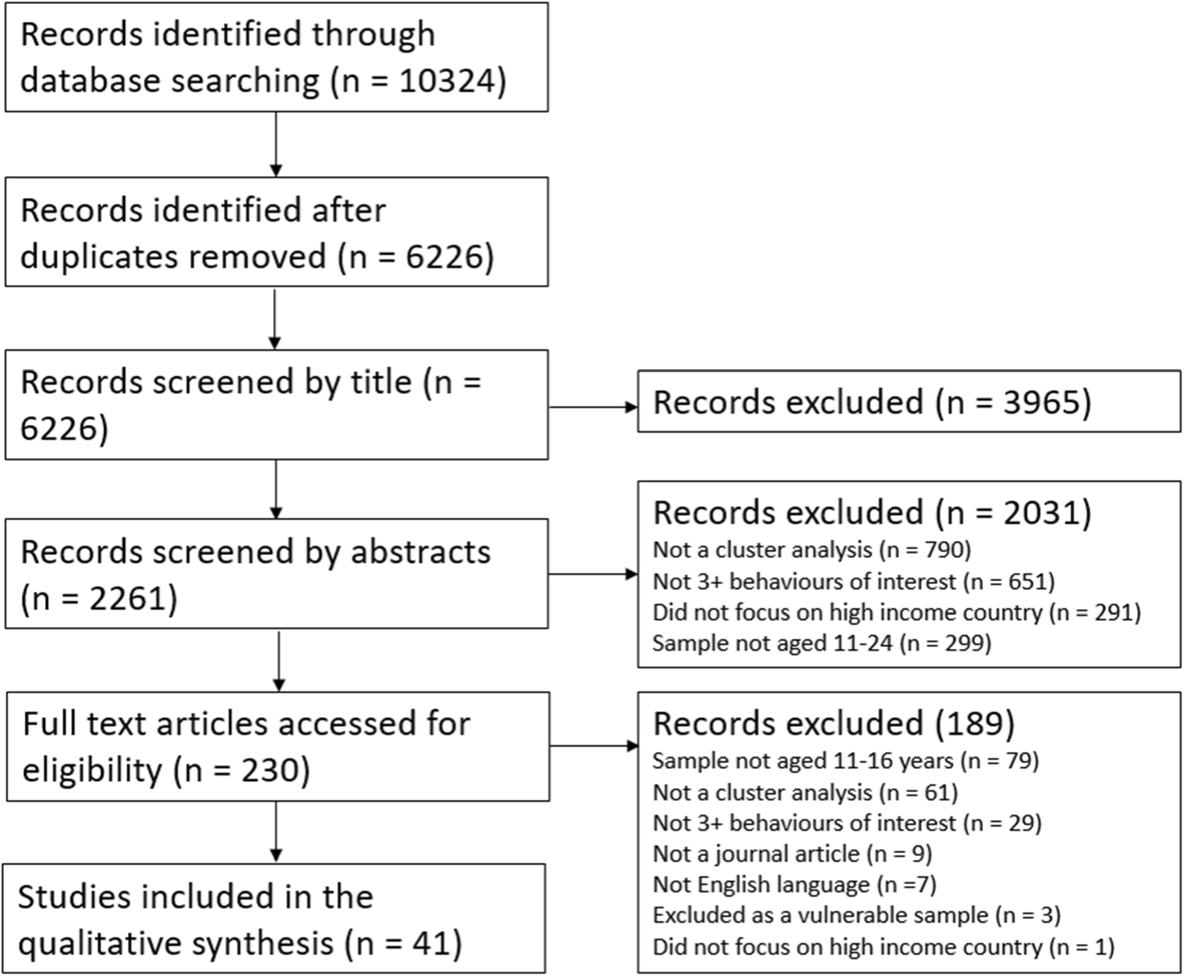
PRISMA diagram

Initial searches returned 6226 potential studies after removal of duplicates. After title, abstract and full text screening, 41 studies were eligible for inclusion (Fig. 1).

### Quality appraisal

Critical appraisal using the AXIS tool did not lead to further exclusions as all papers met the majority of its quality measures. Where papers did not fulfil all of the AXIS quality criteria, they most often lacked detailed information about non-responders (although many studies were secondary data analyses and may have lacked access to this data) (see Supplementary Table 4 for more information). The relative quality of studies was not included in our subsequent analysis but acknowledge the potential for response bias in a number of included studies.

### Study characteristics

Most studies analysed data from North America (*n*=25), predominantly the United States (*n*=22). The remaining studies used data from European countries (*n*=12), South America (n=1) and Australasia (*n*=3). 37 studies were based on general population samples; two studies used socioeconomically deprived samples [21, 22] and two further studies focused on specific ethnic minorities, namely Latino adolescents [23] and a comparison of ‘White American’, ‘American Indian’ and ‘Alaskan natives’ [24]. Sample sizes varied substantially from 234 to 46,283 (M=7754.73, SD=9050.72). Twenty-three studies employed latent class analysis, ten undertook cluster analysis, seven used factor analysis, and one used principal component *and* factor analysis. Three studies reported separate groups for gender [18, 25, 26], two for age [19, 22] and one for ethnicity [27]. Due to this limited sample, no comparisons between sub-groups are undertaken here, the limitations of this are outlined in the discussion. See Supplementary Table 1 for further information on the characteristics of the included studies.

There was heterogeneity in the number and combinations of measured behaviours across studies (Tables 1 and 2). Most studies reported on three (*n*=18) or four behaviours (*n*=12). Alcohol consumption and smoking were the most commonly measured of our behaviours of interest (*n*=40), while gambling was the least commonly measured (*n*=3). The most commonly measured combinations of behaviours were alcohol, smoking and drug use (*n*=16) and those that focused on SNAP (smoking, nutrition, alcohol and physical activity) behaviours (*n*=8). There was also heterogeneity in the measures used to examine each behaviour. For example, alcohol measures included whether the individual had ever drunk alcohol, the frequency of drinking in the last week or last month, the frequency of risky or binge drinking and lifetime drunkenness. We return to this heterogeneity in the discussion.

**Table 1:** The behavioural areas of interest measured by each study

Author	Year	Alcohol	Drug use	Smoking	Physical Activity	Diet	Gambling	Sex
Total		40	30	40	18	19	3	11
Aaro et al. [28]	1995	Y	–	Y	Y	Y	–	–
Ahmadi-Montecalvo et al. [29]	2019	Y	Y	Y	Y	Y	–	Y
Bohnert et al. [21]	2014	Y	Y	Y	–	–	–	–
Burdette et al. [30]	2017	Y	–	Y	Y	Y	–	–
Busch et al. [31]	2013	Y	Y	Y	Y	Y	–	Y
Cardoso et al. [32]	2016	Y	Y	Y	–	–	–	–
Carlerby et al. [33]	2012	Y	–	Y	Y	Y	–	–
Childs & Ray [27]	2015	Y	Y	Y	–	–	–	Y
Connell et al. [34]	2009	Y	Y	Y	–	–	–	–
Conway et al. [35]	2013	Y	Y	Y	–	–	–	–
Delk et al. [19]	2019	Y	Y	Y	–	–	–	–
Dermody et al. [36]	2018	Y	Y	Y	–	–	–	–
Ebin et al. [23]	2001	Y	Y	Y	–	Y	–	Y
Fraga et al. [37]	2011	Y	–	Y	Y	Y	–	–
Hair et al. [38]	2009	Y	Y	Y	Y	–	–	Y
Hasking et al. [39]	2011	Y	Y	Y	–	–	Y	Y
Holund & Rise [40]	1988	Y	–	Y	Y	Y	–	–
Karvonen et al. [41]	2000	Y	–	Y	–	Y	–	–
Kiedrowski & Selya [24]	2019	Y	Y	Y	–	–	–	–
Landsberg et al. [17]	2010	Y	–	Y	Y	Y	–	–
Laxer et al. [42]	2017	Y	Y	Y	Y	Y	–	–
Lazzeri et al. [57]	2018	Y	–	Y	Y	Y	–	–
Lee et al [20]	2019	Y	Y	Y	–	–	–	–
Luk et al. [43]	2012	Y	Y	Y	–	–	–	–
Martínez-Loredo et al. [18]	2019	Y	Y	Y	–	–	Y	–
Mistry et al. [25]	2009	Y	–	Y	Y	Y	–	–
Noel et al. [44]	2013	Y	Y	Y	–	–	–	Y
Neumark-Sztainer et al. [26]	1997	Y	Y	Y	Y	Y	–	Y
Parker et al. [45]	2015	Y	Y	Y	–	–	–	–
Paxton et al. [46]	2007	Y	Y	Y	–	–	–	Y
Pilatti et al. [47]	2013	Y	Y	Y	–	–	–	–
Ranney et al. [48]	2018	Y	Y	Y	–	–	–	–
Rose et al. [22]	2018	Y	Y	Y	–	–	–	–
Russell et al. [49]	2016	Y	Y	Y	Y	Y	–	Y
Su et al. [50]	2018	Y	Y	Y	–	–	–	–
Sullivan et al. [51]	2010	Y	Y	Y	–	–	Y	Y
Theodorakis [52]	2005	–	–	Y	Y	Y	–	–
Turner et al. [53]	2011	Y	–	–	Y	Y	–	–
Van Kooten [54]	2007	Y	Y	Y	Y	Y	–	–
van Nieuwenhuijzen et al. [55]	2009	Y	Y	Y	Y	Y	–	–
White et al. [56]	2013	Y	Y	Y	–	–	–	–

**Table 2:** Combinations of behavioural areas of interest measured by studies

Alcohol	Drug use	Smoking	Physical Activity	Diet	Gambling	Sex	No of Studies	References
**3 Behaviours - 18 studies (44%)**								
Y	Y	Y					15 (37%)	[19–22, 24, 32, 34–36, 43, 45, 47, 48, 50, 56]
		Y	Y	Y			1 (2%)	[52]
Y			Y	Y			1 (2%)	[53]
Y		Y		Y			1 (2%)	[41]
**4 Behaviours – 12 studies (29%)**								
Y		Y	Y	Y			8 (20%)	[17, 25, 28, 30, 33, 37, 40, 57]
Y	Y	Y				Y	3 (7%)	[27, 44, 46]
Y	Y	Y			Y		1 (2%)	[18]
**5 Behaviours – 5 studies (12%)**								
Y	Y	Y	Y	Y			3 (7%)	[42, 54, 55]
Y	Y	Y		Y		Y	1 (2%)	[23]
Y	Y	Y	Y			Y	1 (2%)	[38]
Y	Y	Y			Y	Y	2 (5%)	[39, 51]
**6 Behaviours – 4 studies (10%)**								
Y	Y	Y	Y	Y		Y	4 (10%)	[26, 29, 31, 49]

### Archetypes

The 41 studies contained 198 behavioural clusters, which we grouped into eight archetypes. The first three archetypes are made up of clusters from the 15 studies that solely focus on substance use and no other behavioural risk indicators. These three archetypes are: (1) Poly-Substance Use, (2) Single-Substance Use and (3) Substance Abstainers. The other five archetypes consist of clusters from the 26 studies that examined both substance use and other behavioural risk factors. They are: (4) Substance Use and No or Low Behavioural Risk Indicators, (5) Substance Abstainers and Behavioural Risk Indicators, (6) Complex Configurations, (7) Overall Unhealthy and (8) Overall Healthy. Clusters were allocated to one archetype and there was no overlap between archetypes.

Table 3 details the number of studies reporting clusters within each archetype, the number of clusters within each archetype, and the proportions of primary study participants who belonged to clusters within each archetype. The archetypes vary considerably in terms of the average proportion of primary study populations belonging to their constitutive clusters. The archetypes made up of clusters with the highest average proportion of primary study respondents were Substance Abstainers (average proportion=51%) and Overall Healthy (32%). The archetypes with the lowest average proportion of primary study participants were the apparent highest risk archetypes: Poly-Substance Use (10%) and Overall Unhealthy (10%).

**Table 3:** The number of studies reporting clusters within each archetypes, the number of clusters within each archetype and the average prevalence of respondents in each archetype

Archetypes	Description	Number of Clusters	Number of Studies Reporting Clusters^a^	Average Proportion of Respondents in Clusters (Range)
**1. Poly-Substance Use**	Clusters with engagement in two or more addictive substances.	39 (52%)	15 (100%)	10% (0.2–25%)
**2. Single Substance Use**	Clusters with engagement in one addictive substance.	15 (20%)	9 (60%)	23% (2–80%)
**3. Substance Abstainers**	Clusters reporting no substance use.	21 (28%)	13 (87%)	51% (18–91%)
**Total (substance use only)**		75	15	
**4. Substance Use and No/Low Behavioural Risk Indicators**	Substance use and no/low engagement in majority of risk behavioral indicators measured.	22 (18%)	12 (46%)	23% (4–56%)
**5. Substance Abstainers and Behavioural Risk Indicators**	No substance use and engagement in the majority of behavioural risk indicators measured.	16 (13%)	13 (50%)	28% (6–53%)
**6. Complex Configurations**	May or may not be substance use and where there is no clear majority in terms of the behavioral risk indicators.	23 (19%)	11 (42%)	23% (3–52%)
**7. Overall Unhealthy**	Substance use and engagement in the majority of measured behavioral risk indicators.	29 (24%)	18 (69%)	10% (2–24%)
**8. Overall Healthy**	No substance use and no engagement in the majority of behavioural risk indicators measured.	33 (27%)	18 (69%)	32% (4–85%)
**Total (other studies)**		123	26	
**Total (all studies)**		198	41	–

^a^Only the 15 studies that did not measure any of the non-substance use behavioural risk indicators of interest contribute clusters to archetypes 1–3 whereas only the 26 studies that measured both substance use and other behavioural risk indicators contributed to archetypes 4–8. As such, these values reflect the number of studies that that feasibly could have contributed clusters to each archetypes

Descriptions for each archetype of the number of constituent clusters, the behaviours included in these clusters, and the average proportion of people assigned to constituent clusters follow, with additional information provided in Supplementary Table 2.

#### Poly-substance use

The Poly-Substance Use archetype is constituted by clusters reporting use of multiple substances of interest to this review. All 15 of the studies that focused solely on substance use found at least one cluster that contributed to this archetype, although the number and types of substances used varied substantially across the 39 included clusters. For example, nine clusters involved use of two substances, 19 clusters involved use of three substances and eleven clusters involved use of four or more substances. The majority of clusters involved alcohol, tobacco and cannabis use with (*n*=10) or without (*n*=17) other drugs. The remaining 12 clusters were characterised by use of alcohol and tobacco (*n* = 3), tobacco and cannabis (*n*=2), alcohol and cannabis (*n* = 1), alcohol and other drugs (n = 1), alcohol, tobacco and drugs [1], or some combination of drugs other than cannabis (*n* = 4). The average proportion of study populations in the clusters with the Poly-Substance Use archetype is 10% (range: 0.2–25%). Clusters defined by engagement in a combination of alcohol, tobacco or cannabis use tended to be higher prevalence (range: 3–25%) than those describing engagement with other drugs.

#### Single substance use

Clusters in the Single Substance Use archetype involved use of a single substance of interest. Nine studies contributed 15 clusters and alcohol was the most frequently used substance (*n*=9). The remaining four clusters were characterised by use of methamphetamines (*n*=1), tobacco (*n*=1), cannabis (*n*=1) and other illicit drugs (*n*=1). The alcohol use clusters differed in the measures used, which included ‘any alcohol use’ [32], ‘heavy drinkers’ [47] or ‘binge drinking’ [45]. The proportion of study populations within clusters differed markedly depending on the substance and measure used. Proportions were higher for clusters characterised by tobacco use (14–24%) or very light (80%), light to moderate (16–38%) or heavy (11–14%) alcohol use, when compared to those defined by cannabis use (2–11%), or other illicit drugs (3%).

#### Substance abstainers

The Substance Abstainers archetypes included clusters reporting no use of substances and drew on 21 clusters from 13 of the 15 substance use studies. On average, clusters in the Substance Abstainers archetype accounted for 51% of their respective study populations although this varied substantially (18–91%). The large range is explained partly by several studies having multiple relevant clusters that differ in relation to behaviours beyond our interest areas (e.g. bullying). If the proportion of study samples falling in to the Substance Abstainers archetype is summed within studies contributing multiple clusters, the clusters from these eleven studies account for between 56 and 98% of their respective study samples. The clusters from the remaining studies account for 47% [22] and 20% [47] of the study samples and are slightly older populations (i.e. 14–18 years old, or mean age 15) where we may expect more substance use [58].

#### Substance use and no/low Behavioural risk indicators

Clusters in this archetype are characterised by some substance use and low (or no) engagement in behavioural risk indicators. The archetype comprises 22 clusters contributed by 12 studies, although the number and types of substances used varied substantially between clusters. For example, 11 clusters involved use of one substance, while seven clusters involved use of three or more substances. Most clusters involving use of only one substance were characterised by alcohol use (*n*=10) and clusters involving drug use involved alcohol, tobacco and cannabis use in all but three cases. Twenty of 22 clusters involved engagement in no behavioural risk indicators, with examples of behaviours in the remainder including a medium risk of ‘risky sexual behaviour’ and a low risk of ‘poor diet’ or ‘lack of exercise’ [29]. On average, 23% of primary study samples fell within clusters in this archetype, but this ranged from 4 to 56% due to variation in the number and types of substances used. For clusters defined by the use of alcohol alone the proportion ranged from 21 to 56% [18, 46] where samples also contained older age groups than our core focus of 11–16 year olds) while the proportion was lower for poly-substance use clusters (4–26%), or those with use of tobacco alone (6%).

#### Substance abstainers and Behavioural risk indicators

This archetype consists of 16 clusters from 13 studies, in which young people engaged in all or most behavioural risk indicators measured by the primary study, but abstained from substance use. The majority of clusters are defined in relation to poor diet and exercise (*n*=13), as opposed to engagement in sexual activity and gambling (*n*=3), primarily reflecting how few of the contributing studies measuring sexual activity (*n*=5) or gambling (=2). Sex was also a low prevalence behaviour in three of the five clusters that did include it. The proportion of study populations belonging to clusters in this archetype varied substantially (M=28%, Range: 6–53%), with higher proportions in clusters defined by poor diet and low exercise (18–53% except for [57]) and generally lower proportions in clusters defined by gambling and sexual activity.

#### Complex configurations

Clusters in the Complex Configurations archetype involved contradictory patterns of engagement in behavioural risk indicators (e.g. engagement in both unsafe sex and exercise [38]. Eleven studies examining substance use and behavioural risk indicators contributed 23 clusters to the archetype. Fifteen of these involved substance use, of which 11 involved poly-substance use, mostly alcohol and tobacco, whereas four involved use of a single substance. The eight clusters that did not involve substance use involved contradictory engagement in behavioural risk indicators. For example, the ‘Active snackers’ cluster in Mistry et al. [25] described adolescents who are ‘very unlikely to drink and are non-smoking, but who are all physically active while also having low fruit and vegetable consumption’ [25]. The proportion of young people falling into clusters within this archetype varied substantially from 3 to 53% (M = 23%), and was higher if the cluster did not involve substance use.

#### Overall unhealthy

Clusters in the Overall Unhealthy archetype involved engagement in use of substances and in the majority of measured behavioural risk indicators (although some studies may only include one behavioural risk indicator). The archetype comprises 29 clusters from 18 studies and all but one of these [55] involved poly-substance use - usually alcohol, cigarettes and cannabis (*n* = 14), alcohol tobacco and other drugs (*n* = 7), or alcohol and tobacco (*n* = 5). Within clusters, poly-substance use was accompanied by either multiple behavioural risk indicators (*n* = 16) or a single risk indicator (*n* = 13). Unhealthy diet and low physical activity frequently co-occurred (*n*=10), as did unhealthy diet, low physical activity and sexual activity (*n*=7). Sexual activity was the most common behavioural risk indicator to occur in isolation (*n*=9) followed by gambling (*n*=3). Notably, sex and gambling behaviours were more frequently measured as a single behavioural risk indicator in studies, whereas most studies which measured diet also tended to measure physical activity. The average proportion of individuals falling within clusters in this archetype was 10%. The range was also smaller than for most other archetypes (2–24%).

#### Overall healthy

Clusters in this archetype involved no substance use, and low or no engagement in behavioural risk indicators. The archetype consists of 33 clusters from 18 studies, split between clusters with no substance use or behavioural risk factors (*n*=19) or no substance use and just one behavioural risk indicator (*n*=14). The average proportion of study populations falling within these clusters across all studies was 32%, but the range from 4 to 85% was wider than for any other archetype. As with the Substance Abstainer archetype, this range is explained by studies that identify multiple clusters that we ascribed to the Overall Healthy archetype. When the clusters from single studies contributing multiple clusters to a the archetype are combined, the range narrows to between 42 and 87%, excluding four studies of samples aged 14+ [25, 38, 40, 54], where the prevalence within clusters ranged from 11 to 19%.

## Discussion

This review examined the clustering of a broad range of health-related behaviours in 11–16 year-olds. Eight overarching behavioural archetypes were identified by grouping the clusters described within the primary studies. These archetypes were: (1) Poly-Substance Users, (2) Single Substance Users, (3) Substance Abstainers, (4) Substance Users with No/Low Behavioural Risk Indicators, (5) Substance abstainers with Behavioural Risk Indicators, (6) Complex Configurations, (7) Overall Unhealthy and (8) Overall Healthy.

Our eight overarching archetypes suggest three key findings. First, in the studies included in our review, most 11–16 year-olds fall into one of our ‘healthy’ archetypes which, on average, account for 51% (Substance Abstainers archetype) or 32% (Overall Healthy archetype) of the primary study populations. Second, studies consistently find that small minorities of young people engage in multiple unhealthy behaviours, including polysubstance use, or substance use alongside multiple other risk behaviours, such as having a poor diet, lacking exercise or engaging in sexual activity. These fall into archetypes that account on overage for 10% (Poly-Substance User archetype) and 10% (Overall Unhealthy archetype) of the primary study populations. As would be expected, the proportion of young people in these clusters decreases where greater numbers of substances are used, or when examining heavier use of substances. Third, substantial proportions of young people engage in varied combinations of behaviours (i.e. archetypes 4, 5 and 6) wherein both health promoting and health-risk behaviours co-occur. Young people who engage in health promoting behaviours may, therefore, simultaneously be engaging in other, unhealthy behaviours that counteract any benefits - or vice versa. Importantly, the identified combinations of unhealthy behaviours that young people engage in are diverse and inconsistent across studies. This may present a challenge to the development of effective multi-behavioural health interventions.

### Strengths

This review is the first to examine clustering of health-related behaviours within 11–16 year olds and extends the focus of behaviours considered in other reviews of studies of adult and adolescent populations. A further strength is our development of a new method for synthesis of findings from the heterogeneous literature on behavioural clustering. While our approach does not directly redress the heterogeneity in the literature, it does summarise the key clusters observed in a way that can inform future research, policy and practice. Importantly, this approach facilitated the estimation of the average proportion of individuals falling into similar clusters across multiple studies in this review. Finally, unlike prior reviews which have taken the names of clusters and/or the probabilistic terminology used by primary study authors into account [7–9], our synthesis is based solely on the behaviours measured and numerically standardised probability of engagement in those behaviours.

### Limitations

Drawing data from studies using varied analytical approaches creates problems in comparing results and we are not aware of any available methods for standardising numerical findings from different clustering techniques. Therefore, we used prose, rather than numerical data, to address this problem. However, comparison was still problematic in places as, for example, clusters within archetypes were often characterised by very different levels of engagement in a behaviour, such as light drinking in one cluster and frequent drunkenness in another. In places, this created a false equivalence between different patterns of behaviour that may not have comparable risks of harm.

Despite our age focus, our included studies often included a minority of participants older than 11–16 years. This reflects wide variation in age groups included in primary studies and a decision not to limit our pool of included studies by imposing more rigid age criteria. Nevertheless, the extent and patterns of youth health-related behaviours are known to change across adolescence (for example: [59, 60]; small changes in age foci may therefore result in changes in behavioural clusters, or in the proportions of study samples attributed to them.

We had insufficient data to compare clustering between population subgroups, despite arguments that socioeconomic status, region, age and gender may be important intersections [7, 9, 58, 60]. Studies also came from multiple countries and different time points (ranging from 1982 to 2016), but we did not explore the potential effects of these cultural and temporal specificities. Furthermore, we excluded samples deemed particularly vulnerable to engaging in risky behaviours, such as those young people in the criminal justice system. As such, our conclusions are limited to the general population. We acknowledge that there are demographic groups included in our definition of the ‘general population’, such as those of lower socioeconomic status, wherein prevalence of specific risk behaviours may differ from the general population. However, sub-groups defined, for example, by socioeconomic status account for much larger proportions of the population than, for example, young people in the youth justice system and we elected to include them on this basis. Further analysis of the archetypes which emerge in relation to population sub-groups would therefore be of value.

### Implications for policy and practice

Our behavioural archetypes show that the combinations of health-related behaviours that young people engage in are diverse and complex. Health policy and practice, particularly those advocating multi-behavioural approaches, should therefore be sensitive to such complexity. Specific behavioural clusters identified in individual studies may therefore be insufficiently robust to inform multi-behavioural interventions that are generalizable beyond the context of the original study. In particular, while policy makers and practitioners working in the same context as our primary studies may prefer local evidence, our analysis suggests they should also consider syntheses of broader evidence. This is because researchers’ choices about which behaviours to study and which cluster analysis method to use may also markedly shape findings alongside local factors. While health outcomes were not our focus of attention in constructing archetypes, the complexity we reveal points to a need to determine the clusters associated with greater or lesser risks (or benefits) to health, over time.

### Implications for research

Clustering methods are sensitive to small changes in the data and the measures used: the results of any single study should consequently be treated with caution. To reduce heterogeneity in this literature and maximise comparability across studies, it is important that researchers incorporate similar behaviours and measures in their analyses wherever feasible. Our eight behavioural archetypes can help researchers to think about how to achieve such comparability by suggesting which behaviours commonly cluster (e.g. alcohol, smoking, cannabis use) and which measures provide the most meaningful insight (e.g. measures which differentiate between the level of engagement in different behaviours rather than measures which solely focus on ‘ever use’).

Recognising that researchers will inevitably have their own research interests, we suggest that maximising comparability across studies using different datasets should be prioritised. In other words, where researchers wish to study additional or emerging behaviours (for example, social media use), we suggest these should be added to rather than substitute from a core list of key health-related behaviours. Studies proposing to focus on substance use alone may derive particular benefits from the inclusion of additional behaviours (for example, diet and exercise to avoid the construction of a large ‘abstaining’ cluster which indicates what young people do not do, without additional insight into their health, or the health-related behaviours in which they *do* engage. Further attention should also be given to how behavioural patterns may vary between population sub-groups. In particular, variation in relation to age, gender, socio-economic status and within vulnerable groups are important lines of future enquiry.

## Conclusion

This review identified eight behavioural archetypes that summarise the clustering of health-related behaviours within 11–16 year olds in the included study contexts. These emphasise that behavioural clustering is typically complex and diverse across the adolescent population. Most young people do fall into broadly ‘healthy’ archetypes; however, studies consistently observe that small minorities of adolescents fall into archetypes characterised by heavy substance use and/or multiple risk behaviours.

## Supplementary Information

**Additional file 1.**

## Data Availability

The data supporting the conclusions of this article are included within the article and its additional files.

## Abbreviation

SNAP: Smoking, Nutrition, Alcohol and Physical Activity
