# Supplementary material for: Clustering of health-related behaviours within children aged 11–16: a systematic review
Source: BMC Public Health. 2021 Jan 14;21:137. doi: 10.1186/s12889-020-10140-6 (PMC7807795; doi:10.1186/s12889-020-10140-6)
Supplement: Supplementary file 1 — Additional file 1. [file 12889_2020_10140_MOESM1_ESM.docx]

**Supplementary Table 1: Characteristics of included studies.**

| **Author** | **Year of Publication** | **Country** | **Study design** | **Year data collected** | **Sample Description** | **Sample size** | | **Age** | **Recruitment site** | **Analytical method** | **Number of clusters** |
| --- | --- | --- | --- | --- | --- | --- | --- | --- | --- | --- | --- |
| Aaro et al. | 1995 | Norway | Cross-sectional | 1989-1990 | General population | | 1648 | 15-16 | School-based | Confirmatory factor analysis | 2 |
| Ahmadi et al. | 2019 | United States | Cross-sectional | 2013 | General population | | 13583 | 14-19 | School-based | Latent class analysis | 5 |
| Bohnert et al. | 2014 | United States | Cross-sectional | 2007-2009 | Low SES | | 1416 | 12-18 | Federal Qualified Community Health Centres | Latent class analysis | 3 |
| Burdette et al. | 2017 | United States | Cross-sectional | 1995 | General population | | 7827 | 12-18 | School-based | Latent class analysis | 4 |
| Busch et al. | 2013 | Holland | Cross-sectional | 2012 | General population | | 2690 | 11-18 | School-based | Two step cluster analysis | 4 |
| Cardosa et al. | 2016 | United States | Cross-sectional | n/a^1^ | General population | | 1036 | 11-19 | School-based | Latent class analysis | 6 |
| Carlerby et al. | 2012 | Sweden | Pooled repeat cross-sectional | 1997-2006 | General population | | 11972 | 11-16 | School-based | K-mean cluster analysis | 5 |
| Childs & Ray | 2015 | United States | Cross-sectional | 1995-1996 | General population | | 13568 | 13-17 | In home interviews | Latent class analysis | 7 |
| Connell et al. | 2009 | United States | Cross-sectional | 2005 | General Population | | 13953 | 14-18 | School-based | Latent class analysis | 4 |
| Conway et al. | 2013 | United States | Cross-sectional | 2010 | General population | | 2254 | 15-16 | School-based | Latent class analysis | 4 |
| Delk et al. | 2019 | United States | Cross-sectional | 2015-2016 | General population | | 2733 | 12-17 | School-based | Latent class analysis | 9 |
| Dermody et al. | 2018 | United States | Cross-sectional | 2005 | General Population | | 15607 | 14-18 | School-based | Latent class analysis | 4 |
| Ebin et al. | 2001 | United States | Cross-sectional | n/a | Latino Adolescents | | 609 | 11-19 | Health Clinics | Exploratory factor analysis | 4 |
| Fraga et al. | 2011 | Portugal | Longitudinal | 2003-2004 | General population | | 1612 | 13-14 | School-based | Wards average cluster analysis | 3 |
| Hair et al. | 2009 | United States | Cross-sectional | 2002 | General population | | 4586 | 16-17 | School-based | Latent class analysis | 4 |
| Hasking et al. | 2011 | Australia | Cross-sectional | n/a | General population | | 548 | 12-17 | School-based | Latent class analysis | 3 |
| Holund & Rise | 1988 | Denmark | Cross-sectional | 1982-1985 | General population | | 234 | 14 | School-based | Factor analysis | 4 |
| Karvonen et al. | 2000 | Finland | Cross-sectional | 1992-1993 | General population | | 280 | 16 and 18 year olds | Home-based | K-means cluster analysis | 3 |
| Kiedrowski & Selya | 2019 | United States | Cross-sectional | 2013 | White, American Indian and Alaska Natives | | 46283 | 14-18 | School-based | Factor analysis | 7 |
| Landsberg et al. | 2010 | Germany | Pooled cross-sectional | 2004-2006 | General population | | 1894 | 14 | School-based | Cluster analysis | 3 |
| Laxer et al. | 2017 | Canada | Longitudinal | 2012-2013 | General population | | 30147 | 14-17 | School-based | Latent Class analysis | 4 |
| Lazzerri et al. | 2018 | Italy | Cross-sectional | 2010 | General population | | 3291 | 11-15 | School-based | Cluster analysis | 6 |
| Lee et al. | 2019 | United States | Cross-sectional | 2015 | General population | | 15624 | 14-18 | School-based | Latent class analysis | 5 |
| Luk et al. | 2012 | United States | Cross-sectional | 2005-2006 | General population | | 7508 | 11-16 | School-based | Latent class analysis | 4 |
| Martínez‑  Loredo et al. | 2019 | Spain | Cross-sectional | n/a | General population | | 1644 | 14-15 | School-based | Latent class analysis | 9 |
| Mistry et al. | 2009 | United States | Cross-sectional | 2003 | General population | | 4010 | 14 | School-based | Cluster analysis | 8 |
| Neumark-Sztainer et al. | 1997 | United States | Cross-sectional | 1986-87 | General population | | 25868 | 12-20 | School-based | Factor analysis | 9 |
| Noel et al. | 2013 | New Zealand | Cross-sectional | n/a | General population | | 9107 | 12-19 | School-based | Latent class analysis | 4 |
| Parker et al. | 2015 | United States | Cross-sectional | 2013 | General population | | 18680 | 14-19 | School-based | Latent class analysis | 5 |
| Paxton et al. | 2007 | United States | Cross-sectional | 2003 | General population | | 13973 | 12-17 | School-based | K-means clusters | 11 |
| Pilatti et al. | 2013 | Argentina | Cross-sectional | n/a | General population | | 583 | 13-18 | School-based | Latent class analysis | 5 |
| Ranney et al. | 2018 | United States | Cross-sectional | n/a | General population | | 5001 | 12-17 | Emergency department based^2^ | Latent class analysis | 3 |
| Rose et al. | 2018 | United States | Longitudinal^3^ | 2014 | Rural population | | 4822 | 11-18 | School-based | Latent class analysis | 8 |
| Russell et al. | 2016 | Canada | Cross-sectional | 2009-2010 | General population | | 10261 | 14-16 | School-based | Principal component and factor analysis | 4 |
| Su et al. | 2018 | United States | Cross-sectional | 2009 | General population | | 9155 | 13-17 | School-based | Latent class analysis | 4 |
| Sullivan et al. | 2010 | United States | Cross-sectional |  | General population | | 2549 | 14-18 | School-based | Latent class analysis | 4 |
| Theodorakis | 2005 | Greece | Cross-sectional | n/a | General population | | 3307 | 10-16 | School-based | Cluster analysis | 4 |
| Turner et al. | 2011 | Canada | Cross-sectional | 2005 | General population | | 445 | 14-17 | School-based | Two step cluster analysis | 3 |
| van Kooten | 2007 | Holland | Cross-sectional | n/a | General population | | 5730 | 12-16 | School-based | Factor analysis | 4 |
| van Nieuwenhuij-zen et al. | 2009 | Holland | Cross-sectional | 2005-6 | General population | | 504 | 12-15 | Mixed: telephone, face to face and online surveys | Factor analysis | 2 |
| White et al. | 2013 | Australia | Cross-sectional | 2007 | General population | | 1402 | 12-17 | National Household Survey | Latent class analysis | 3 |

^1^n/a where date of data collection is not specified ^2^Admission for any reason ^3^Only wave 4 was used

Supplementary Table 2: Full list of clusters included in each archetype and their study of origin.

| Archetype | Study | Cluster Description |
| --- | --- | --- |
| 1. **Poly-Substance Use** |  |  |
|  | Bohnert et al. (2014) | **Polysubstance user^1^** (11%) May smoke cigarettes, all drink alcohol, likely heavy episodic drinking, likely cannabis use, likely cannabis consequences, unlikely prescription drug use and very unlikely illegal drug use. |
|  | Bohnert et al. (2014) | **Cannabis user** (25%) May smoke cigarettes, unlikely to use alcohol, likely to smoke cannabis, no illegal drug use and very unlikely prescription drug use. |
|  | Cardoso et al. (2016) | **High polysubstance use** (4%) All alcohol use, may smoke cigarettes, all high use of cannabis and may use illicit drugs. |
|  | Cardoso et al. (2016) | **Low polysubstance use** (4%) All alcohol use, unlikely tobacco use, all cannabis use and unlikely illicit drug use |
|  | Connell et al. (2009) | **Occasional polysubstance users (23%)** All used alcohol in the last month and likely to have drank heavily, may have smoked, unlikely to have tried cannabis, cocaine, inhalants or other hard drugs. |
|  | Connell et al. (2009) | **Frequent polysubstance users (13%)** Very likely to have drank in last month and likely to have drank heavily, likely to have smoked cigarettes and used cannabis and may have used cocaine, inhalants and other hard drugs. |
|  | Conway et al. (2013) | **Predominant polysubstance users** (8%) All cannabis users, likely medication misuse, maybe other illicit drugs, likely smoking, all drinking alcohol, very likely binge drinking. |
|  | Conway et al. (2013) | **Predominant alcohol users** (23%) Maybe cannabis use, very unlikely medication use, very unlikely other illicit drugs, maybe smoking, all drinking alcohol, likely binge drinking. |
|  | Delk et al. (2019) | **All product use** (10%) Very likely to have used cigarettes and e-cigarettes. All used alcohol and likely to have used cannabis. |
|  | Dermody et al. (2018) | **Alcohol Users (13%)** All use alcohol and may binge drink, may use e-cigarettes, unlikely to use cannabis and very unlikely to use other tobacco products. |
|  | Dermody et al. (2018) | **Marijuana/ Nicotine Users (6%)** May use cannabis and e-cigarettes. Unlikely to use alcohol, cigarettes and other tobacco products. |
|  | Dermody et al. (2018) | **Polysubstance/ecig users (5%)** All use alcohol and cannabis and likely to binge drink and use e-cigarettes, unlikely to use cigarettes and other tobacco products. |
|  | Dermody et al. (2018) | **Polysubstance/tobacco users (8%)** All use alcohol and very likely to binge drink. Likely to use cannabis, cigarettes. e-cigarettes and other tobacco products. |
|  | Kiedrowski & Selya (2019) | **Factor 1** (n not provided) May have tried steroids, all used injected drugs. No use of cigarettes, alcohol, cannabis or other drugs. |
|  | Kiedrowski & Selya (2019) | **Factor 2** (n not provided) Likely to have used cocaine, heroin and ecstasy. May have used inhalants and methamphetamine. No use of cigarettes, alcohol, cannabis or other drugs. |
|  | Kiedrowski & Selya (2019) | **Factor 3** (n not provided) Likely to smoke, may have used alcohol and cannabis. No use of other drugs. |
|  | Kiedrowski & Selya (2019) | **Factor 2** (n not provided) Likely to have used heroin and injected drugs. May have used steroids, unlikely to have used inhalants. No use of alcohol, cigarettes, cannabis or other drugs. |
|  | Kiedrowski & Selya (2019) | **Factor 3** (n not provided) Likely to smoke and use cannabis, may have used alcohol. No use of other drugs. |
|  | Kiedrowski & Selya (2019) | **Factor 4** (n not provided) Likely to have used ecstasy, may have used cocaine. No use of alcohol, cigarettes, cannabis or other drugs. |
|  | Lee et al. (2019) | **First-step social experimenter** (25%) Unlikely to smoke cigarettes but may use electronic vape. Likely to drink alcohol and may use cannabis. Very unlikely to use inhalants, synthetic cannabis or prescription drugs. No other substance use. |
|  | Lee et al. (2019) | **Second-step social experimenter** (0.6%) Likely to smoke, use electronic vapes and take prescription drugs. Very likely to smoke and use cannabis. May use cocaine, synthetic cannabis and ecstasy. Unlikely to use inhalants. Very unlikely to use methamphetamines. No heroin, steroids or injected illegal drugs. |
|  | Lee et al. (2019) | **Pill experimenter** (0.4%) May drink and use prescription drugs. Unlikely to smoke cigarettes or an electronic vape or to take cannabis, cocaine, inhalants, ecstasy or synthetic cannabis. Very unlikely to use methamphetamines or illegal injected drugs. No heroin. |
|  | Lee et al. (2019) | **Full experimenter** (0.2%) Very likely to smoke cigarettes, drink alcohol, use cocaine and methamphetamines. Likely to use an electronic vape, use cannabis, inhalants, heroin, ecstasy, synthetic cannabis, steroids, prescription drugs and injected illegal drugs. |
|  | Luk et al. (2012) | **Substance-using bullies** (5%) May smoke, use cannabis and cyber-bully. All drink alcohol and verbally bully. Very likely to be drunk and socially exclude. Likely to physically bully and spread rumours. |
|  | Luk et al. (2012) | **Substance users** (19%) May smoke, all drink alcohol, Likely to be drunk, Unlikely to use cannabis. |
|  | Parker et al. (2015) | **Moderate polysubstance use** (10%) All alcohol use, all binge drinking, likely marijuana use, may use cigarettes, may use cigars/cigarellos, unlikely prescription drug use. |
|  | Parker et al. (2015) | **High poly substance use** (6%) All alcohol use, all binge drinking, all marijuana use, all cigarette use, all cigar/cigarellos use and very likely prescription drug use. |
|  | Parker et al. (2015) | **Alcohol and marijuana use** (8%) May use alcohol, no binge drinking, likely marijuana use, may use cigarettes, may use cigars/cigarellos and unlikely prescription drug use. |
|  | Pilatti et al. (2013) | **Risky polysubstance abusers** (24%) High levels of frequent drinking, high alcohol quantity consumption, moderate levels of drunkenness, moderate levels of tobacco use and moderate levels of drug use. |
|  | Ranney et al. (2018) | **Moderate risk group** (5%) All smoked a cigarette in the past year, may consume alcohol and cannabis. No other drug use. |
|  | Ranney et al. (2018) | **High risk** (4%) Very likely to consume alcohol, very likely to use cannabis and all have used other drugs. Very likely to use cigarettes. |
|  | Rose et al. (2018) | **Initiators high school** (6%) Very likely to have drunk alcohol 1-5 times. No non-drinkers or more frequent alcohol use. All smoked cigarettes 1-5 times. Very likely to have used cannabis 1-5 times, no more frequent cannabis use, very unlikely to have never used cannabis. May have never used inhalants, unlikely to have used inhalants 1-5 times, very unlikely to have used inhalants 6-19 times, no more frequent use of inhalants. May have never misused prescription drugs, may have misused prescription drugs 1-5 times, very unlikely to have misused prescription drugs 6-19 times, no more frequent misuse of prescription drugs. |
|  | Rose et al. (2018) | **Initiators - moderate-to-high-frequency high school** (10%) May have drunk alcohol 1-5 times, unlikely to have drunk 6-19 times, very unlikely to have drunk 20+ times and no non-drinkers. May have smoked cigarettes 1-5 times, unlikely to have smoked cigarettes 6-19 times and very unlikely to have smoked cigarettes 20+ times or not at all. May have used cannabis 1-5 times, unlikely to have used cannabis 6-19 times or 20+ times and very unlikely to never have used cannabis. Likely to be non-users of inhalants, very unlikely to have used inhalants 1-5, 6-19, or 20+ times. May have never misused prescription drugs, unlikely to have misused prescription drugs 1-5 times and very unlikely to have misused prescription drugs 6-19 or 20+ times. |
|  | Rose et al. (2018) | **Initiators middle school** (4%) Very likely to have drunk alcohol 1-5 times, no more frequent use of alcohol, very unlikely to have never drunk alcohol, all smoked cigarettes 1-5 times, very likely to use cannabis 1-5 times, no more frequent use of cannabis, very unlikely to have never used cannabis, may have never used inhalants, unlikely to have used inhalants 1-5 times, very unlikely to have used inhalants 6-19 times, no more frequent use of inhalants, may have never misused prescription drugs, unlikely to have misused prescription drugs 1-5 times, no more frequent misuse of prescription drugs. |
|  | Rose et al. (2018) | **Initiators - moderate-to-high-frequency middle school** (3%) All have drunk alcohol 1-5 times. May have smoked cigarettes 1-5 times, unlikely to have smoked cigarettes 6-19 times, very unlikely to have never smoked cigarettes or smoked 20+ times. May have used cannabis 1-5 times, unlikely to have used cannabis 6-19 times or 20+ times, very unlikely to have never used cannabis. May have never used inhalants, unlikely to have used inhalants 1-5 times, very unlikely to have used inhalants 6-19 times or 20+ times. May have never misused prescription drugs, unlikely to have misused prescription drugs 1-5 times and very unlikely to have misused prescription drugs 6-19 time or 30+ times. |
|  | Su et al. (2018) | **ACM users** (14%) Likely to have smoked cigarettes, very likely to have drank alcohol, likely to use cannabis, very unlikely to have used other illegal drugs. |
|  | Su et al. (2018) | **Poly users** (4%) All smoked cigarettes, may have used snuff or chewing tobacco, all drink alcohol, all use cannabis, very likely to have used other illegal drugs, may have used inhalants, all used non-prescription drugs to get high and very likely to have used prescription drugs to get high. |
|  | White et al. (2013) | **Limited range drug users** (18%) Likely to use tobacco, all use alcohol, may use cannabis, very unlikely to use ecstasy and no use of any other substance. |
|  | White et al. (2013) | **Extended range drug users** (2%) All use tobacco, all use alcohol, all use cannabis, likely to use ecstasy, may use amphetamines, may use pain killers, unlikely to use cocaine, unlikely to use hallucinogens, unlikely to use tranquilisers and unlikely to use inhalants. |
| 1. **Single Substance Use** |  |  |
|  | Cardoso et al. (2016) | **Illicit drug use** (3%) No alcohol use, unlikely tobacco use, no cannabis use and all use illicit drugs. |
|  | Cardoso et al. (2016) | **Cannabis use** (2%) No alcohol use, very unlikely tobacco use, all cannabis use and very unlikely illicit drug use. |
|  | Cardoso et al. (2016) | **Alcohol use** (16%) All alcohol use, very unlikely tobacco use, no cannabis use and very unlikely illicit drug use. |
|  | Conway et al. (2013) | **Predominant cannabis users** (11%) Maybe cannabis, unlikely medication misuse, unlikely other illicit drugs, unlikely smoking, no drinking alcohol, very unlikely binge drinking. |
|  | Delk et al. (2019) | **Tobacco ever use** (24%) Very likely to have used e-cigarettes and likely to have used cigarettes. Unlikely to have drunk alcohol or smoked cannabis. |
|  | Delk et al. (2019) | **Tobacco ever use** (14%) Very likely to have used cigarettes and e-cigarettes. Unlikely to have used alcohol and cannabis. |
|  | Kiedrowski & Selya (2019) | **Factor 1** (n not provided) All used methamphetamine, no use of alcohol, cigarettes, cannabis or other drugs. |
|  | Parker et al. (2015) | **Alcohol use and binge drinking** (14%) All alcohol use, may binge drink, unlikely to use marijuana, very unlikely to use cigarettes, no use of cigars/cigarellos and very unlikely prescription drug use. |
|  | Pilatti et al. (2013) | **Moderate to high drinkers** (26%) High levels of frequent drinking, moderate alcohol quantity consumption, no drunkenness, low levels of drug use and low levels of tobacco use. |
|  | Pilatti et al. (2013) | **Light drinkers** (21%) Moderate frequency of drinking, moderate alcohol quantity consumption, no drunkenness, very low levels of tobacco use, and no drug use. |
|  | Pilatti et al. (2013) | **Heavy drinkers** (11%) High levels of frequent drinking, high quantities of alcohol consumed, moderate drunkenness, no tobacco use and no drug use. |
|  | Rose et al. (2018) | **Primary alcohol users high school** (38%) Likely to have used alcohol 1-5 times in lifetime, unlikely to have never drank alcohol, likely to have never smoked cigarettes, unlikely to have smoked cigarettes 1-5 times in lifetime, may have never used cannabis, unlikely to have used cannabis 1-5 times in lifetime, very likely to have never used inhalants or misused prescription drugs, very unlikely to have used inhalants or misused prescription drugs 1-5 times in lifetime. No more frequent use of alcohol, inhalants, cigarettes or prescription drugs than 1-5 times over lifetime, very unlikely to have used cannabis 6-19 times over lifetime and no use of cannabis 20 or more times during lifetime. |
|  | Rose et al. (2018) | **Primary alcohol users middle school** (31%) May have drunk alcohol 1-5 times over lifetime, may never have drunk alcohol, likely never to have smoked cigarettes and unlikely to have smoked cigarettes 1-5 times over lifetime, likely to have never used cannabis, very unlikely to have used cannabis 1-5 times in lifetime, likely to have never used inhalants, unlikely to have used inhalants 1-5 times in lifetime, likely to have never misused prescription drugs, very unlikely to have misused prescription drugs 1-5 times in lifetime. No more frequent use of any substances than 1-5 times in lifetime. |
|  | Su et al. (2018) | **ALC Users** (26%) Unlikely to smoke cigarettes, likely to have drunk beer or wine, likely to have drunk hard liquor. Unlikely to use cannabis, no other drug use. |
|  | White et al. (2013) | **Sometimes alcohol users** (80%) May use alcohol, no tobacco use and no drug use. |
| 1. **Substance Abstainers** |  |  |
|  | Bohnert et al. (2014) | **Non-user** (65%) No use of alcohol, prescription drugs, illegal drugs or tobacco. |
|  | Cardoso et al. (2016) | **No risk** (71%) No alcohol use, very unlikely tobacco use, no cannabis use and no illicit drug use. |
|  | Connell et al. (2009) | **Non Users (27%)** No alcohol, cigarette, cannabis, cocaine, inhalant or other hard drug use. |
|  | Connell et al. (2009) | **Alcohol Experimenters (38%)** Unlikely to have drank alcohol in the last month, no use of cigarettes, cannabis, cocaine, inhalant or other hard drug use. |
|  | Conway et al. (2013) | **Non-users** (59%) No cannabis, no medication misuse, no other illicit drugs, no smoking, very unlikely drinking alcohol, no binge drinking. |
|  | Delk at al. (2019) | **No risk** (78%) No use of tobacco products, alcohol or cannabis. |
|  | Delk at al. (2019) | **No risk** (48%) No use of tobacco products, alcohol or cannabis. |
|  | Delk at al. (2019) | **No risk** (40%) No cannabis use or cigarettes, very unlikely to have drank alcohol in the last 30 days or to have ever tried an e-cigarette. |
|  | Delk et al. (2019) | **Tobacco susceptible** (22%) Very unlikely to have used cannabis or alcohol. Unlikely to have used cigarettes or e-cigarettes. |
|  | Delk et al. (2019) | **Tobacco susceptible** (38%) Very unlikely to have tried cigarettes. Unlikely to have used e-cigarettes or drank in the last month. No use of cannabis. |
|  | Delk et al. (2019) | **Tobacco susceptible** (38%) Very unlikely to have tried cigarettes. Unlikely to have used e-cigarettes or drank in the last month. No use of cannabis. |
|  | Dermody et al. (2018) | **Non Users (68%)** Very unlikely to use alcohol and e-cigarettes, no cannabis, cigarettes, tobacco. |
|  | Lee et al. (2019) | **Abstinent** (64%) Very unlikely to have used alcohol. No cigarettes, electronic vape, cannabis or other drugs. |
|  | Luk et al. (2012) | **Non-involved** (58%) No smoking, being drunk, cannabis use, physical bullying, rumour spreading or cyber bullying. Very unlikely to drink alcohol, socially exclude or to verbally bully. |
|  | Luk et al. (2012) | **Bullies** (18%) No smoking, being drunk or using cannabis, Unlikely to drink alcohol or cyber-bully. May physically bully and spread rumours. Very likely to verbally bully. Likely to socially exclude. |
|  | Parker et al. (2015) | **No use** (63%) Very unlikely alcohol use, no binge drinking, very unlikely marijuana use, no cigarette use, no cigars/cigarello use and no prescription drug use. |
|  | Pilatti et al. (2013) | **Substance use naives** (20%) No alcohol, tobacco or drug use. |
|  | Ranney et al. (2018) | **Low risk** (91%) Very unlikely to use cannabis and drink. No smoking or other drug use. |
|  | Rose et al. (2018) | **Substance nonusers high school** (47%) All have never used alcohol, smoked cigarettes, used cannabis, used inhalants, or misused prescription drugs. |
|  | Rose et al. (2018) | **Substance nonusers middle school**: (63%) All have never used alcohol, smoked cigarettes, taken inhalants, used cannabis or misused prescription drugs. |
|  | Su et al. (2018) | **Abstainers** (56%) No cigarettes, alcohol, cannabis, illegal drugs or prescription drugs. |
| 1. **Substance Use and No/Low Behavioural Risk Indicators** |  |  |
|  | Ahmadi et al. (2019) | **Alcohol drinkers** (15%) High likelihood of alcohol and cannabis use, medium risk of other drugs, low risk of smoking. Low risk of poor diet and lack of physical exercise. Medium risk of risky sexual behaviour, violence, and unhealthy weight control. Low risk of suicidal thoughts. |
|  | Burdette et al. (2017) | **Moderate risk with substance use** (15%) All eat breakfast and very likely to have eaten two portions of fruit/veg yesterday, very likely to have exercised 3 times in the past week, may have smoked in the last month, likely to have binge drunk in the last month. |
|  | Burdette et al. (2017) | **High risk** (21%) May eat breakfast and have eaten at least two portions of fruit and vegetables yesterday, may have exercised three times in last week, likely to have smoked, may binge drink. |
|  | Childs & Ray (2019) | **High delinquent/experimenters** (7%) Low alcohol use, medium use of cannabis and cigarettes, low sexual activity – low likelihood of multiple sexual partners, high likelihood of violence, theft, public disorder and status offending. |
|  | Fraga et al. (2011) | **Cluster 2** (52%) Likely to be a non-smoker, no never drinkers, very likely to experiment with alcohol but unlikely to consider themselves a drinker. Unlikely to never exercise, very unlikely to exercise between once a month and between once a week, may exercise 2-6 times a week, unlikely to exercise every day, eats a moderate amount of fruit. |
|  | Hair et al. (2009) | **Moderate risk group one** (21%) Unlikely to engage in delinquency, unlikely to engage in smoking, unlikely to engage in drug use, unlikely to engage in unsafe sex, may drink alcohol in risky ways, and likely to exercise. |
|  | Hasking et al. (2011) | **Rule breakers** (51%) No smoking, may drink alcohol, very unlikely to have taken illegal drugs, no gambling and no underage sex. |
|  | Hasking et al. (2011) | **Minor delinquents** (41%) Very unlikely to smoke cigarettes, likely to drink alcohol, unlikely to take illegal drugs, very unlikely to regularly gamble, unlikely to have had sexual intercourse. |
|  | Landsberg et al. (2010) | **High activity and medium-risk behaviours** (35%) High structured and unstructured physical activity, lower media time, likely active commute to school, may have healthy or mixed diet, not risk-related diet, may be monthly drinker and very unlikely to be smokers. |
|  | Martínez‑Loredo et al. (2019) | **Smokers with alcohol abuse** (18%) No non-drinkers. May have used alcohol 10-19 times or > 20 times in last year, very unlikely to have used alcohol 1-2, 3-5 or 6-9 times in the last year. May have smoked tobacco more than 20 times in the last year, unlikely to have never smoked tobacco or smoked 1-2 times in the last year, very unlikely to have smoked tobacco 3-5, 6-9 or 10-19 times in the last year. Unlikely to have never used cannabis, used cannabis 1-2 times in the last year or used cannabis 20+ times in the last year, very unlikely to have used cannabis 3-5, 6-9 or 10-19 times in the last year. Very unlikely to gamble. |
|  | Martínez‑Loredo et al. (2019) | **Non-users** (54%) May have drunk alcohol in the last year. Unlikely to have drunk 1-2 times and very unlikely to have drank more than 3 times. No smoking cigarettes or cannabis. Very unlikely to bet on sports and no other gambling. |
|  | Martínez‑Loredo et al. (2019) | **Non-users** (56%) May have used alcohol in the last year. Unlikely to have drunk 1-2 or 3-5 times in last year, very unlikely to have drank 6-9 times and no drinking 10+ times. No smoking or cannabis. Very unlikely to have played bingo and no other gambling activity. |
|  | Martínez‑Loredo et al. (2019) | **Smokers with alcohol abuse girls** (16%) No non-drinkers. Likely to have drunk alcohol 20+ times in the past year. Unlikely to have drunk alcohol 3-5, 6-9 or 10-19 times in the last year. No non-smokers, likely to have smoked more than 20+ times in the last year. Unlikely to have smoked 3-5 times and very unlikely to have smoked 6-9 or 10-19 times. Unlikely to have never used cannabis and to have used it 1-2, 3-5 or 20+ times in last year. Very unlikely to have used cannabis 6-9 or 10-19 times. No gambling activity. |
|  | Martínez‑Loredo et al. (2019) | **Exclusively alcohol users girls** (13%) All drinkers, may drink 10-19 times a year. May have not smoked in the last year or smoked 1-2 times. Likely to not have used cannabis in the last year. Very unlikely to gamble. |
|  | Noel et al. (2013) | **Risky** (12%) May have alcohol problems, unlikely to use cannabis weekly, may use cigarettes, may have risky motor vehicle behaviour, unlikely to have unsafe sex. |
|  | Noel et al. (2013) | **Distressed** (6%) Very likely to be depressed, may attempt suicide, unlikely to have alcohol problems, no weekly cannabis use, may use cigarettes, may have risky motor vehicle use, unlikely to have unsafe sex, unlikely to engage in violence and unlikely to be a delinquent. |
|  | Paxton et al. (2007) | **Rode with drinker and light substance user** (8%) May have driven with someone drinking, may use alcohol, may use tobacco and unlikely to have had sex. No cannabis or illicit drug use. |
|  | Paxton et al. (2007) | **Frequent alcohol abusers** (5%) All high alcohol use, all risky binge drinking, very likely to use tobacco, likely low cannabis use, and likely low sexual risk. No illicit drug use. |
|  | Paxton et al. (2007) | **Illicit substance users** (4%) All use illicit drugs and may use IV drugs, very likely to have low levels of tobacco use, likely to use low levels of alcohol, likely to have low cannabis use and low sexual risk. |
|  | Paxton et al. (2007) | **Moderately violent/aggressive alcohol users** (6%) Likely to carry a weapon, very likely to engage in fighting, very likely to engage in low levels of alcohol use. No sexual risk. No tobacco, cannabis or illicit substance use. |
|  | Russell et al. (2016) | **Gateway substance use** (26%) Likely to drink alcohol, may use cannabis and cigarettes. Unlikely to have used other illicit drugs. No physical activity. Unlikely to eat fast food. Very unlikely to have unprotected sex. |
|  | Turner et al. (2011) | **Active, high screen time users** (32%) Moderate levels of physical activity, moderate fruit and vegetable consumption and moderate alcohol consumption. High levels of television viewing, high levels of internet use. |
| 1. **Substance Abstainers and Behavioural Risk Indicators** |  |  |
|  | Ahmadi et al. (2019) | **Low risk** (53%) Lowest risk of smoking, drinking, risky sexual behaviour and drug use. Medium risk of poor diet and sedentary behaviour. Lowest risk of violence, depressive symptoms, suicidal thoughts and unhealthy weight control. |
|  | Busch et al. (2013) | **Bully behaviour** (no n presented) No smoking, drug use, alcohol or sex. Very likely to bully and be a victim of bullying. No healthy nutrition or physical activity. |
|  | Busch et al. (2013) | **Problematic screen time use** (no n presented) No smoking, drug use, alcohol or sex. No healthy nutrition or physical activity. Likely to compulsively play videogames, may compulsively use the internet. |
|  | Childs & Ray (2019) | **Sexual risk takers black** (6%) Low use of cannabis, cigarettes and alcohol, high likelihood of sex with multiple partners. Low likelihood of violence, theft, public disorder and status offending. |
|  | Holund & Rise (1988) | **Sugar behaviour** (18%) All frequently consume sugar. Very unlikely to drink alcohol and exercise. No smoking. |
|  | Karvonen et al. (2000) | **Mixed cluster** (32%) High likelihood of poor diet, high likelihood of abstaining from alcohol and not smoking. |
|  | Lazzeri et al. (2018) | **Quasi-healthy group** (8%) Low alcohol, smoking, physical activity and healthy diet. |
|  | Martínez‑Loredo et al. (2019) | **Exclusively gamblers boys** (18%) May be non-drinkers in last year, unlikely to have drunk alcohol 1-2 or 3-5 times in the last year, very unlikely to have drunk alcohol 6-9, 10-19 or 20+ times in the last year. Very likely to have not smoked in the last year. No cannabis use. May bet on sports, play the lottery and use scratch cards. Unlikely to play bingo and poker, very unlikely to use electronic gaming machines and no casino games. |
|  | Mistry et al. (2009) | **Sedentary snackers boys (**22%) All report low physical activity and very likely to report low fruit/veg consumption. No smoking and very unlikely to drink alcohol. |
|  | Mistry et al. (2009) | **Sedentary snackers girls** (25%) All report low physical activity and likely to report low fruit/veg consumption. No alcohol or smoking. |
|  | Neumark-Sztainer et al. (1997) | **School-related behaviours girls** (no n provided) No alcohol or cannabis use, unlikely sexual activity and tobacco use, very unlikely healthy eating and no exercise. |
|  | Sullivan et al. (2010) | **Experimenters (36%)** Likely to have had sex, may have gambled. Likely to never have drank alcohol or used cannabis. Very unlikely to have smoked cigarettes. |
|  | Theodorakis et al. (2005) | **Deficient health profile** (38%) Low exercise, fruit consumption and smoking. |
|  | Turner et al. (2011) | **Less active, least frequent drinkers** (22%) very low levels of physical activity, low fruit and vegetable consumption and very low alcohol consumption. |
|  | Van Kooten (2007) | **Sedentary behaviours** (51%) No tobacco, cannabis and alcohol use. No fruit and veg intake. No physical activity. Likely to use a PC and to watch television. |
|  | Van Kooten (2007) | **Sweet consumption** (31%) Likely to drink soft drinks and very likely to consume sweets. No smoking, drinking and cannabis use. No physical activity. |
| 1. **Complex Configurations** |  |  |
|  | Carlerby et al. (2012) | **Drunkenness** (7%) All drunkenness, no smoking, no wish to lose weight, unlikely low physical activity, very unlikely inadequate tooth-brushing, may have low vegetable consumption, unlikely high soft-drink consumption. |
|  | Carlerby et al. (2012) | **Multiple risk behaviours** (6%) Likely drunkenness, all smoking, very unlikely wish to lose weight, unlikely low physical activity, unlikely inadequate tooth brushing, likely low vegetable consumption, may have high soft-drink consumption. |
|  | Ebin et al. (2001) | **Factor 2** (n not provided) Unlikely to be sexually active, smoke cigarettes or drink alcohol. May use cannabis. Very unlikely to eat breakfast. |
|  | Ebin et al. (2001) | **Factor 1** (n not provided) Likely cigarette use, likely alcohol use and may use cannabis. No sex, no eating breakfast. |
|  | Fraga et al. (2011) | **Cluster 3** (3%) All smoke, unlikely to never drink and may experiment with alcohol, may exercise 2-6 times a week, lower fruit consumption. |
|  | Hair et al. (2009) | **High risk group** (27%) May engage in delinquency, likely to smoke, likely to use drugs, likely to drink in risky ways, may engage in unsafe sex and may exercise. |
|  | Hair et al. (2009) | **Moderate risk group two** (37%) Very unlikely to engage in drug use, very unlikely to engage in risky drinking, unlikely to smoke, unlikely to engage in unsafe sex, and likely not to exercise. |
|  | Landsberg et al. (2010) | **Low activity and low-risk behaviour** (39%) Lower structured and unstructured physical activity, medium media time, likely active commute to school, unlikely healthy or risk-related diet, may have mixed diet, no alcohol or smoking. |
|  | Landsberg et al. (2010) | **High media time and high-risk behaviour** (26%) Lower structured and higher unstructured physical activity, higher media time, likely active commute to school, unlikely healthy or risk-related diet, may have mixed diet, all monthly drinkers, may be smokers. |
|  | Laxer et al. (2017) | **Traditional School Athletes** (no n provided) May exercise, likely to eat breakfast but may eat fast food and have low fruit and vegetable intake. Very unlikely to binge drink, smoke and use cannabis. |
|  | Laxer et al. (2017) | **Inactive screenagers** (no n provided) May exercise, very likely to have low fruit and vegetable intake and likely to eat fast food. May eat breakfast. May have a lot of screen time. Very unlikely to binge drink, smoke and use cannabis. |
|  | Lazzeri et al. (2018) | **Symptomatic group** (31%) High alcohol consumption and moderate smoking. High experience of somatic and psychological symptoms (e.g. headache, nervousness). Low physical activity and moderate healthy diet. |
|  | Lazzeri et al. (2018) | **Violent group** (10%) Moderate alcohol consumption and smoking. High physical activity and low healthy eating. High rates of violence. |
|  | Lazzeri et al. (2018) | **Smoking drinkers group** (30%) High scores for both alcohol consumption and smoking. Low physical activity and moderate healthy diet. |
|  | Lazzeri et al. (2018) | **Non-drinking, smokers group** (11%) High score for smoking, low score for alcohol drinking, high physical activity and low healthy eating. |
|  | Mistry et al. (2009) | **Risk takers boys** (7%) Very likely to smoke and may drink alcohol. May report low fruit/veg consumption but unlikely to report low physical activity. |
|  | Mistry et al. (2009) | **Active snackers boys** (52%) All physically active, all have low fruit/veg consumption, very unlikely to drink, no smoking. |
|  | Mistry et al. (2009) | **Risk takers girls** (16%) All alcohol users and likely have low fruit/veg consumption. Unlikely to smoke and to report low physical activity. |
|  | Mistry et al. (2009) | **Active snackers girls** (43%), All physically active, all have low fruit/veg consumption, no smoking or alcohol. |
|  | Sullivan et al. (2010) | **Non sexually active, high risk behaviour youth** (5%) Very likely to have never had sex. May have gambled, used cannabis, drank alcohol and smoked cigarettes in the last year. |
|  | Sullivan et al. (2010) | **Abstainers** (36%) Very likely to have never had sex and to have not used cannabis, drank alcohol and smoked cigarettes in the last year. May have gambled. |
|  | Theodorakis et al. (2005) | **Deviant smoking profile** (12%) Low exercise, high smoking, moderate fruit consumption and violence. |
|  | Turner et al. (2011) | **Active, low screen time users** (46%) Moderate physical activity, low fruit and vegetable consumption and low alcohol consumption. Low tv viewing, low internet use, low homework, low talking with friends. |
| 1. **Overall Unhealthy** |  |  |
|  | Aaro et al. (1995) | **Addictive behaviours** (no n provided) Likely to smoke and drink alcohol. Unlikely to eat healthy food. No physical activity. |
|  | Ahmadi et al. (2019) | **Depressed and suicidal** (14%) Medium cigarette, drug and alcohol use, medium likelihood of risky sex, high chance of poor diet and sedentary behaviour. High chance of depressive thoughts, suicidal ideation and bullying. |
|  | Ahmadi et al. (2019) | **Tobacco and alcohol users** (9%) Highest likelihood of smoking. High risk of alcohol and drug use. Medium risk of risky sexual activity and poor diet. |
|  | Ahmadi et al. (2019) | **Highest risk/multi-risk behaviour** (9%) High risk of smoking, alcohol drinking, drug use, unhealthy weight control, violence and risky sex. Medium risk of poor diet and high risk of sedentary behaviour. |
|  | Busch et al. (2013) | **Risk prone behaviour** (no n presented) Likely to smoke cigarettes, binge drink and smoke cannabis. Likely to have had intercourse. No healthy nutrition or physical activity. |
|  | Childs & Ray (2019) | **High risk, diverse behaviour black** (2%) Highest use of alcohol, cannabis and cigarettes. Medium likelihood of sex with multiple partners, high likelihood of violence, theft, public disorder and status offending. |
|  | Childs & Ray (2019) | **High delinquency and substance use white** (5%) Highest use of alcohol and cannabis, high use of cigarettes, high likelihood of multiple sexual partners and low likelihood of never have had sex, high likelihood of violence, theft, public disorder and status offending. |
|  | Childs & Ray (2019) | **Experimenters white** (16%) Low cannabis use, high use of cigarettes, medium use of alcohol, medium likelihood of multiple sexual partners. |
|  | Hasking et al. (2011) | **Major delinquents** (8%) May smoke cigarettes, likely to drink alcohol, may regularly gamble, may take illegal drugs, likely to have had underage sexual intercourse. |
|  | Holund & Rise (1988) | **Problem behaviour** (5%) May smoke and drink alcohol. Very unlikely to exercise. Unlikely to have a regular diet and to consume sugar. |
|  | Karvonen et al. (2000) | **Unhealthy cluster** (24%) Low likelihood of abstaining from alcohol and not smoking. Likely to have a poor diet. |
|  | Lazzeri et al. (2018) | **Screen time** (11%) Moderate alcohol consumption and high smoking. Low physical activity and low healthy diet, high screen time. |
|  | Laxer et al. (2017) | **Moderately active substance users** (no n provided) May exercise, likely to have low fruit and vegetable intake, to miss breakfast and very likely to eat fast food. Likely to smoke, use cannabis and binge drink. |
|  | Martínez‑Loredo et al. (2019) | **Broad users boys** (10%). All drinkers, very likely to have drank 20+ times in the last year. Very unlikely to be non-smokers, and likely to have smoked 20+ times in the last year. Likely to have used cannabis and may have used 20+ times in the last year. May play poker, electronic gaming machines, lottery, scratch cards and bet on sports. Unlikely to play bingo or casino games. |
|  | Martínez‑Loredo et al. (2019) | **Broad users girls** (7%) All drinkers, may have drank 20+ times in the last year. All smokers, may have smoked 20+ times in the last year. Likely to have used cannabis in the last year. May play lottery and use scratch cards but unlikely to play bingo, poker, bet on sports, and use electronic gambling machines. Very unlikely to play casino games. |
|  | Martínez‑Loredo et al. (2019) | **Alcohol and gambling users girls** (9%) Very unlikely to be a non-drinker or to have drank alcohol 10-19 times in last year. Unlikely to have drank alcohol 1-2 times, 3-5 or 6-9 times and no drinking 20+ times in last year. Very likely to have not used tobacco in the last year. No cannabis use in last year. May gamble via bingo, sports betting, scratch cards, likely to do the lottery but no poker or other casino games. Unlikely to use electronic gaming machines. |
|  | Neumark-Sztainer et al. (1997) | **Risk-taking behaviours boys** (no n provided) Likely tobacco, alcohol and cannabis use. Likely sexual activity, no exercise, very unlikely unhealthy eating. |
|  | Neumark-Sztainer et al. (1997) | **Risk taking behaviours girls** (no n provided) Likely tobacco, alcohol and cannabis use, may engage in sexual activity, very unlikely unhealthy eating and no exercise. |
|  | Noel et al. (2013) | **Multiple** (5%) Very likely to have alcohol problems, likely to have weekly cannabis use, very likely cigarette use. |
|  | Paxton et al. (2007) | **Comprehensive extreme risk takers** (3%) All illicit substance use, all alcohol use, all tobacco use, all risky sexual intercourse. |
|  | Paxton et al. (2007) | **Cannabis users** (9%) All cannabis use, very likely to have moderate sexual risk, very likely to moderately use tobacco and alcohol. |
|  | Paxton et al. (2007) | **Comprehensive moderate risk takers** (3%) All high levels of cannabis and alcohol use, all moderate levels of sexual risk. No illicit substance use. |
|  | Paxton et al. (2007) | **Substance abusers and sexual risk takers** (5%) All use high levels of alcohol, all high levels of tobacco, all high levels of binge drinking, all high levels of illicit substance use, very likely to have moderate sexual risk. |
|  | Paxton et al. (2007) | **Alcohol and sexual risk takers** (14%) All have moderate risky sexual behaviour, very likely to use low levels of alcohol, likely to have low levels of tobacco use, may have low levels of cannabis use. No other illicit drugs. |
|  | Russell et al. (2016) | **Hard drugs and weapons** (7%) Likely to have used illicit drugs. May have used prescription drugs, cannabis and cigarettes. May have friends who carry weapons. Unlikely to drink alcohol or binge drink. May have had unprotected sex. Unlikely to eat fast food. No exercise. |
|  | Sullivan et al. (2010) | **High diverse, risk behaviour youth** (22%) Likely to have had sex, gambled, drank alcohol and used cannabis. May have smoked cigarettes. |
|  | Van Kooten (2007) | **Addictive behaviours** (19%) Likely to use tobacco, cannabis and drink alcohol. No fruit and veg intake. No physical activity. |
|  | Van Nieuwenhuijzen et al. (2009) | **Alcohol young adolescents** (n not provided) Very likely to consume higher numbers of glasses of alcohol per day, and to consume alcohol on great number of days per week. Very likely to take drugs. May smoke. No physical activity, no indicators of healthy diet. |
|  | Van Nieuwenhuijzen et al. (2009) | **Delinquency young adolescents** (n not provided) May smoke, unlikely to have a healthy diet and to exercise. No alcohol and no drugs. Very likely delinquency in the last year. |
| 1. **Overall Healthy** |  |  |
|  | Aaro et al. (1995) | **Health-enhancing behaviours** (no n provided) Unlikely to exercise, may eat healthy food and exhibit safety seeking behaviours. No smoking, alcohol, or unhealthy food. |
|  | Burdette et al. (2017) | **Low Risk** (32%) Very likely to eat breakfast and likely to have eaten two portions of fruit/veg yesterday, likely to have exercised 3 times in the past week, very likely not to have smoked in the last month, no binge drinking in the last month. |
|  | Burdette et al. (2017) | **Moderate risk with inactivity** (32%) Likely to eat breakfast and to have eaten two portions of fruit/veg yesterday, may have exercised three times in the last week, very likely not to have smoked, no binge drinking. |
|  | Busch et al. (2013) | **Sedentary behaviour** (no n presented) May have a healthy diet, likely to exercise. No smoking, alcohol, sex or drug use. Likely to excessively play computer games, may watch TV excessively. |
|  | Carlerby et al. (2012) | **Wish to lose weight** (22%) No drunkenness, no smoking, unlikely wish to lose weight, unlikely low physical activity, may be low vegetable consumption, very unlikely high soft-drink consumption. |
|  | Carlerby et al. (2012) | **Low risk behaviour** (51%) No drunkenness, no smoking, unlikely low physical activity, may have low vegetable consumption, very unlikely high soft-drink consumption. |
|  | Carlerby et al. (2012) | **Inadequate tooth-brushing** (14%) No drunkenness, no smoking, no wish to lose weight, unlikely low physical activity, all inadequate tooth-brushing, likely low vegetable consumption, unlikely high soft-drink consumption. |
|  | Childs & Ray (2019) | **Low risk/abstainers black** (85%) Low likelihood of alcohol, cigarettes and cannabis. High likelihood of never having had sex and low likelihood of having had multiple sexual partners. |
|  | Childs & Ray (2019) | **Low risk/abstainers white** (79%) Low likelihood of alcohol, cigarette and cannabis. High likelihood of never having had sex and low likelihood of having multiple sexual partners. |
|  | Ebin et al. (2001) | **Factor 4** (n not provided) Very likely to eat breakfast. No cigarettes, very unlikely to drink alcohol, unlikely to smoke cannabis or have sex. |
|  | Ebin et al. (2001) | **Factor 3** (n not provided) All use vitamins. No cigarettes, alcohol, cannabis or sex. Unlikely to eat breakfast. |
|  | Fraga et al. (2011) | **Cluster 1** (46%) Very likely never to have smoked, very unlikely to be an experimental smoker, never a smoker, all never drinkers, unlikely to never take part in sports, very unlikely to exercise between once a month and between once a week, may exercise 2-6 times a week, unlikely to exercise every day. |
|  | Hair et al. (2009) | **Low risk group** (16%) No behaviours of delinquency, no smoking, no drug use, no risky drinking, no unsafe sex and may exercise. |
|  | Holund & Rise (1988) | **Spare time activity** (4%) May engage in physical activity. Very unlikely to smoke cigarettes, unlikely to drink alcohol. Unlikely to have a regular diet and to consume sugar. |
|  | Holund & Rise (1988) | **Regularity of dietary behaviour** (12%) May have regular diet. Very unlikely to smoke cigarettes, drink alcohol or consume sugar. No physical activity. |
|  | Karvonen et al. (2000) | **Healthy cluster (44%)** Higher chance of better diet, abstinence from alcohol and non smoking. |
|  | Laxer et al. (2017) | **Health conscious (no n provided)** May exercise, likely to eat breakfast and to avoid fast food but may have low fruit and vegetable intake. Very unlikely to binge drink, smoke and use cannabis. |
|  | Mistry et al. (2009) | **Salutary adherents boys** (19%) No alcohol, no cigarettes, no low physical activity and no low fruit/vegetable consumption. |
|  | Mistry et al. (2009) | **Salutary adherents girls** (17%) No alcohol, no cigarettes, no low physical activity and no low fruit/vegetable consumption. |
|  | Neumark-Sztainer et al. (1997) | **Exercise boys** (no n provided) No tobacco or cannabis use, very unlikely alcohol use. Unlikely frequent dieting, to engage in sexual activity. No unhealthy eating or binge eating. Likely to exercise. |
|  | Neumark-Sztainer et al. (1997) | **Quietly disturbed behaviours boys** (no n provided) No tobacco, alcohol use or cannabis use. Very unlikely sexual activity and unhealthy eating and no exercise. May binge eat, frequently diet, vomit and attempt suicide. |
|  | Neumark-Sztainer et al. (1997) | **Health promoting behaviours boys** (no n provided) No tobacco, alcohol or cannabis use. No sexual activity, no unhealthy eating but very unlikely to exercise. |
|  | Neumark-Sztainer et al. (1997) | **School-related behaviours boys** (no n provided) Very unlikely tobacco use, no alcohol or cannabis use. Very likely to drop out and to get school grades lower than C’s. Unlikely sexual activity, very unlikely unhealthy eating and no exercise. |
|  | Neumark-Sztainer et al. (1997) | **Quietly disturbed behaviours girls** (no n provided) No tobacco or cannabis. Very unlikely alcohol, sexual activity and unhealthy eating. Unlikely to exercise. Likely binge eating, frequent dieting and vomiting. May attempt suicide. |
|  | Neumark-Sztainer et al. (1997) | **Health-promoting behaviours girls** (no n provided) No tobacco, alcohol or cannabis use, no sexual activity, no unhealthy eating or binge eating. Very unlikely to exercise or frequently diet. |
|  | Noel et al. (2013) | **Healthy** (78%) No alcohol, cannabis, sex. Very unlikely to smoke cigarettes. |
|  | Paxton et al. (2007) | **Low level risk takers** (36%) likely to have low levels of alcohol use, may have low levels of tobacco use, unlikely to use other illicit drugs or have sexual risk. |
|  | Paxton et al. (2007) | **Non-risk takers** (9%) No cannabis, drugs, alcohol, smoking or sexual risk. |
|  | Russell et al. (2016) | **Overt risk-taking** (6%) Unlikely to drink alcohol, binge drink, smoke cigarettes or smoke cannabis. Unlikely to have had unprotected sex. No physical activity. No fast food. May engage in other risky behaviours. |
|  | Russell et al. (2016) | **Physical activity** (5%) Likely to participate in higher rates of school sanctioned physical activity, non-school physical activity and physical activity in a typical week. Very unlikely to drink alcohol and smoke cigarettes. No drug use, fast food or unprotected sex. |
|  | Theodorakis et al. (2005) | **Mainstream healthy profile** (43%) High exercise and fruit consumption. Low smoking and violence. |
|  | Theodorakis et al. (2005) | **Deviant violent profile** (7%) High exercise, fruit consumption and violence. Low smoking. |
|  | Van Kooten (2007) | **Health-enhancing behaviours** (42%) More likely to eat fruit, more likely to eat vegetables, may do physical activity. No tobacco use, no alcohol use, no cannabis use. |
|  |  |  |

^1^ Where additional behaviours informed the titles given to clusters by primary study authors, such behaviours have been left in the description of clusters within Supplementary Table 2 (for example, the ‘deviant violent profile’ cluster in the overall healthy archetypes, otherwise only behaviours of interest are reported.

**Supplementary Table 3: Search Strategy**

1. Clustering terms
   - Cluster Analysis/ Principal Component Analysis/Factor Analysis, Statistical/
   - Cluster* or co-occur* or latent or typolog* or principal component analysis or factor analysis or concurrence or mixture models
2. Population terms
   - ADOLESCENT/ CHILD/ STUDENTS/ Peer Group/ Young Adult/
   - youth or adolescen* or child* or young adult or teen* or young people or student* or schoolchild* or school-age* school-child* or school child* or peer or college or university or school
3. Behavioural categories
   - HEALTH BEHAVIOR/ LIFE STYLE/ ADOLESCENT BEHAVIOR/ BEHAVIOR, ADDICTIVE /
   - health behaviour or health behavior or lifestyle or life-style or life style or health risk or health-risk or adolescent behavior or adolescent behavior or youth behaviour or youth behavior or addictive behaviour or addictive behavior.
4. Specific behaviours
   - Smoking tobacco
     - SMOKING WATER PIPES/ or WATER PIPE SMOKING/ or SMOKING/ or TOBACCO SMOKING/ or CIGAR SMOKING/ or SMOKING, NON-TOBACCO products/ or CIGARETTE SMOKING/ or SMOKING CESSATION/ or SMOKING REDUCTION/ or SMOKING PIPES/ or PIPE SMOKING/ Tobacco smokeless/ Vaping/ or Nicotine/ or Electronic Nicotine Delivery System/
     - smok* or tobacco* or e-cigar* or vap* or SNUS or cigar* or nicotine
   - Alcohol
     - Alcohol Drinking/ Alcoholism/ Alcohol Abstinence/
     - Alcohol*, or alcohol drinking or heavy episodic drinking or binge drinking or alcohol abstinence or non-drinking or risky drinking or drink*
   - Diet and Exercise
     - EXERCISE/ SEDENTARY LIFESTYLE/ Weight Lifting/
     - HEALTHY DIET/ or DIET FAD/ or DIET, HIGH-FAT/ or DIET/ or DIET, SODIUM-RESTRICTED/ or DIET, FOOD AND NUTRITION/ or DIET, FAT-RESTRICTED/, or DIET, VEGAN/, or DIET, VEGETARIAN/ or DIET, HIGH-PROTEIN/ or DIET, HIGH-PROTEIN LOW-CARBOHYDRATE/ or DIET, CARBOHYDRATE-RESTRICTED Carbonated beverages/ or dietary sucrose/FRUIT/ VEGETABLES/
     - diet or exercise or fruit or vegetable* or salt or fat or sugar or vegan* or vegetarian* or low-carb or low carb or carb-restricted or carb restricted or high-protein or high protein or low-protein or low protein or physical activity or physical inactivity or sedentary or weight train* or weight-train* or weight-lift* or weightlift* or weight train* or strength-train* or strength train*
   - Drugs
     - Narcotics/ SUBSTANCE-RELATED DISORDERS/ HEROIN DEPENDENCE/ or HEROIN/ Analgesics, Opioid/ OPIUM DEPENDENCE/ or OPIUM/ or Opioid-Related Disorders/ COCAINE RELATED DISORDERS/ or COCAINE/ or CRACK-COCAINE/ or COCAINE SMOKING/ CANNABIS/ Psychotropic drugs/ or PHENCYCLIDINE ABUSE/ or PHENCYCLIDINE/ Benzodiazepines/ or Benzodiazepinones Marijuana Abuse/ or Barbiturates/ AMPHETAMINES/ METHAMPHETAMINE/ N-Methyl-3, 4-methylenedioxyamphetamine/ HALLUCINOGENS/ KETAMINE/ Lysergic Acid Diethylamide/ INHALANT ABUSE/ Solvents/ SUBSTANCE ABUSE, INTRAVENOUS/ Street Drugs/ Prescription Drugs/ or Nonprescription drugs/ PRESCRIPTION DRUG MISUSE/ STEROID/
     - ((narcotic or heroin or opiate* or opioid* or opium or cocaine or cannabis or marijuana or marihuana or hash* or phencyclidine or PCP or benzodiaz* or amphetamine* or methamphetamine* or MDMA or ecstasy or hallucinogen* or LSD or ketamine or inhalant* or glue or solvent or substance* or new psychoactive substance or popper or gas or mephedrone or magic mushroom or stimulant or tranquiliser or methadone or psychedelics or GHB or gammahydroxybutrate or GBL or gamma-butyrolactone or steroid or class A or illegal drug* or illicit drug* or prescription drug* or street drug* or non-prescription drug* or nonprescription drug* or performance enhancing drug*) adj (abus* or misus* or us* or problem or depend* or addict* or disorder*)).ti,ab
   - Other addictive, or otherwise relevant, youth behaviours
     - GAMBLING/
     - SEX/ or SAFE SEX/ or UNSAFE SEX/
     - Video Games/
     - INTERNET/
     - Social Media/

(sex* or risky sexual behaviour or risky sexual behavior or safe sex or unsafe sex or gambl* or internet* or screen* or social media or facebook or twitter or myspace or bebo or reddit or youtube or whatsapp or facebook messenger or webchat or QQ or QZone or Doujin or Tik Tok or Weibo or Instagram or tumblr or Viber or Social network* or SNS or Web* or Google+ or Vine or Pinterest or Snapchat or gaming or online gam* or video gam* or online activity).ti,ab

**Supplementary Table Four: Results of quality appraisal using AXIS tool.**

| **Author** | **1. Were the aims/objectives of the study clear?** | **2. Was the study design appropriate for the stated aim(s)?** | **3. Was the sample size justified?** | **4. Was the target/reference population clearly defined? (Is it clear who the research was about?)** | **5. Was the sample frame taken from an appropriate population base so that is closely represented the target/reference population under investigation?** | **6. Was the selection process likely to select subjects/participants that were representative of the target/reference population under investigation** | **7. Were measures undertaken to address and categorise non-responders?** | **8. Were the risk factor and outcome variables measured appropriate to the aims of the study?** | **9. Were the risk factor and outcome variables measured correctly using instruments/measurements that had been trialled, piloted or published previously?** | **10. Is it clear what was used to determine statistical significance and/or precision estimates? (p-values, confidence intervals)** | **11. Were the methods (including statistical methods) sufficiently described to enable them to be repeated?** | **12. Were the basic data adequately described?** | **13. Does the response rate raise concerns about non-response bias?** | **14. If appropriate, was information about non-responders described?** | **15. Were the results internally consistent?** | **16. Were the results presented for all the analyses described in the methods?** | **17. Were the authors discussions and conclusions justified by the results?** | **18. Were the limitations of the study discussed?** | **19. Were there any funding sources or conflicts of interest that may affect the authors interpretation of the results?** | **20. Was ethical approval or consent of participants attained?** |
| --- | --- | --- | --- | --- | --- | --- | --- | --- | --- | --- | --- | --- | --- | --- | --- | --- | --- | --- | --- | --- |
| Aaro et al. | Y | Y | Y | Y | Y | Y | N | Y | Y | Y | Y | Y | N | Y | Y | Y | Y | N | N | Y |
| Ahmadi et al. | Y | Y | Y | Y | Y | Y | Y | Y | Y | Y | Y | Y | N | N | Y | Y | Y | Y | N | Y |
| Bohnert et al. | Y | Y | Y | Y | Y | Y | Y | Y | Y | Y | Y | Y | N | Y | Y | Y | Y | Y | N | Y |
| Burdette et al. | Y | Y | Y | Y | Y | Y | Y | Y | Y | Y | Y | Y | N | Y | Y | Y | Y | Y | N | Y |
| Busch et al. | Y | Y | Y | Y | Y | Y | N | Y | Y | N | Y | Y | N | Y | Y | Y | Y | Y | N | Y |
| Cardoso et al. | Y | Y | Y | Y | Y | Y | N | Y | Y | Y | Y | Y | Not known | N | Y | Y | Y | Y | N | Y |
| Carlerby et al. | Y | Y | Y | Y | Y | Y | Y | Y | Y | Y | Y | Y | N | Y | Y | Y | Y | Y | N | Y |
| Childs & Ray | Y | Y | Y | Y | Y | Y | N | Y | Y | Y | Y | Y | N | N | Y | Y | Y | Y | N | Y |
| Connell et al. | Y | Y | Y | Y | Y | Y | N | Y | Y | Y | Y | N | N | N/A | Y | Y | Y | Y | N | Y |
| Conway et al. | Y | Y | Y | Y | Y | Y | N | Y | Y | Y | Y | Y | N | N | Y | Y | Y | Y | N | Y |
| Delk et al. | Y | Y | Y | Y | Y | Y | Y | Y | Y | Y | Y | Y | N | N | Y | Y | Y | Y | N | Y |
| Dermody et al. | Y | Y | Y | Y | Y | Y | N | Y | Y | Y | Y | Y | N | N/A | Y | Y | Y | Y | N | Y |
| Ebin et al. | Y | Y | Y | Y | Y | Y | N | Y | Y | Y | Y | Y | N | N | Y | Y | Y | Y | N | Y |
| Fraga et al. | Y | Y | Y | Y | Y | Y | Y | Y | Y | Y | Y | Y | N | Y | Y | Y | Y | Y | N | Y |
| Hair et al. | Y | Y | Y | Y | Y | Y | Y | Y | Y | Y | Y | Y | Y | Y | Y | Y | Y | Y | N | Y |
| Hasking et al. | Y | Y | Y | Y | Y | Y | N | Y | Y | Y | Y | Y | Not known | N | Y | Y | Y | Y | N | Y |
| Holund & Rise | Y | Y | Y | Y | Y | Y | N | Y | Y | N | Y | Y | Not known | N | Y | Y | Y | N | N | Y |
| Karvonen et al. | Y | Y | Y | Y | Y | Y | N | Y | Y | N | Y | Y | N | N/A | Y | Y | Y | Y | N | Y |
| Kiedrowski & Selya | Y | Y | Y | Y | Y | Y | N | Y | Y | Y | Y | Y | Y | N | Y | Y | Y | Y | N | Y |
| Landsberg et al. | Y | Y | Y | Y | Y | Y | N | Y | Y | Y | Y | Y | Y | N | Y | Y | Y | Y | N | Y |
| Laxer et al. | Y | Y | Y | Y | Y | Y | Y | Y | Y | Y | Y | Y | N | Y | Y | Y | Y | Y | Y | Y |
| Lazerri et al. | Y | Y | Y | Y | Y | Y | N | Y | Y | Y | Y | Y | Not known | N | Y | Y | Y | Y | N | Y |
| Lee et al. | Y | Y | Y | Y | Y | Y | Y | Y | Y | Y | Y | Y | N | N | Y | Y | Y | Y | N | Y |
| Luk et al. | Y | Y | Y | Y | Y | Y | N | Y | Y | Y | Y | Y | N | N | Y | Y | Y | Y | N | Y |
| Martinez-Loredo et al. | Y | Y | Y | Y | Y | Y | N | Y | Y | Y | Y | Y | Not known | N | Y | Y | Y | Y | N | Y |
| Mistry et al. | Y | Y | Y | Y | Y | Y | N | Y | Y | Y | Y | Y | Y | N | Y | Y | Y | Y | N | Y |
| Neumark-Sztainer et al | Y | Y | Y | Y | Y | Y | N | Y | Y | Y | Y | Y | Not known | N | Y | Y | Y | Y | N | Y |
| Noel et al. | Y | Y | Y | Y | Y | Y | N | Y | Y | Y | Y | Y | N | N | Y | Y | Y | Y | N | Y |
| Parker et al. | Y | Y | Y | Y | Y | Y | N | Y | Y | Y | Y | Y | Not known | N | Y | Y | Y | Y | N | Y |
| Paxton et al. | Y | Y | Y | Y | Y | Y | N | Y | Y | Y | Y | Y | Y | N | Y | Y | Y | Y | N | Y |
| Pilatti et al. | Y | Y | Y | Y | Y | Y | N | Y | Y | Y | Y | Y | Not known | N | Y | Y | Y | Y | N | Y |
| Ranney et al. | Y | Y | Y | Y | Y | Y | N | Y | Y | Y | Y | Y | N | N | Y | Y | Y | Y | N | Y |
| Rose et al. | Y | Y | Y | Y | Y | Y | N | Y | Y | Y | Y | Y | Not known | N | Y | Y | Y | Y | N | Y |
| Russell et al. | Y | Y | Y | Y | Y | Y | N | Y | Y | N | Y | Y | N | N | Y | Y | Y | Y | N | Y |
| Su et al. | Y | Y | Y | Y | Y | Y | N | Y | Y | Y | Y | Y | Not known | N | Y | Y | Y | Y | N | Y |
| Sullivan et al. | Y | Y | Y | Y | Y | Y | N | Y | Y | Y | Y | Y | N | N | Y | Y | Y | Y | Y | Y |
| Theodorakis et al. | Y | Y | Y | Y | Y | Y | N | Y | Y | Y | Y | Y | Not known | N | Y | Y | Y | Y | N | Y |
| Turner et al. | Y | Y | Y | Y | Y | Y | Y | Y | Y | Y | Y | Y | N | N | Y | Y | Y | Y | N | Y |
| van Kooten et al. | Y | Y | Y | Y | Y | Y | Y | Y | Y | Y | Y | Y | N | Y | Y | Y | Y | Y | N | Y |
| van Nieuwenhuijzen et al. | Y | Y | Y | Y | Y | Y | N | Y | Y | Y | Y | Y | N | N | Y | Y | Y | Y | N | Y |
| White et al. | Y | Y | Y | Y | Y | Y | Y | Y | Y | Y | Y | Y | Not known | N | Y | Y | Y | Y | N | Y |
